# Decoding plant volatile stress signals across scales: from molecular responses to ecosystem dynamics

**DOI:** 10.3389/fpls.2026.1815183

**Published:** 2026-06-02

**Authors:** Matthew Chidozie Ogwu, Rehema Ulimboka, Olugbemiga Ojo Aliu

**Affiliations:** 1 Goodnight Family Department of Sustainable Development, Living Learning Center, Appalachian State University, Boone, NC, United States; 2 Department of Biology, The University of Dodoma, Dodoma, Tanzania; 3 Department of Environmental Biology, Auchi Polytechnic, Auchi, Edo State, Nigeria

**Keywords:** biosensing technologies, ecosystem monitoring, herbivore-induced plant volatiles, multi-scale ecological signaling, plant–plant communication, precision agriculture, stress signaling

## Abstract

Plant volatile organic compounds (VOCs) represent one of the most dynamic and integrative biochemical signaling systems linking molecular plant stress responses to ecosystem-level processes. This review provides an integrative cross-scale framework for understanding the biochemical pathways, regulatory networks, ecological functions, and technological applications of stress-induced volatile emissions. At the molecular and cellular levels, VOC emissions are regulated through complex enzymatic and hormonal pathways involving jasmonates, salicylates, ethylene, and abscisic acid, enabling plants to respond rapidly to abiotic and biotic stressors such as drought, herbivory, temperature extremes, salinity, and atmospheric pollution. These volatile signals extend beyond individual plants, functioning as mediators of plant–plant communication, plant–microbe interactions, and multi-trophic ecological networks that shape community dynamics and ecosystem resilience. Recent technological advancements, including mass spectrometry platforms, remote sensing systems, biosensors, and artificial intelligence–driven analytical frameworks, have transformed the ability to detect, interpret, and predict stress-induced VOC emissions in real time. Integrating these technologies with multi-omics datasets and digital twin modeling enables the development of predictive monitoring systems capable of scaling plant stress detection from agricultural fields to regional ecosystems. Despite these advances, significant challenges remain, including variability in emission profiles across species and environments, atmospheric transformation of volatile signals, methodological inconsistencies, and limitations in large-scale monitoring infrastructure. Future research should focus on establishing global networks for monitoring plant volatiles, standardized measurement protocols, and integrated biosensing infrastructures that can link plant stress signals to Earth-system observations. Decoding plant volatile stress signaling across scales offers a transformative pathway to advance climate-resilient agriculture, biodiversity conservation, and predictive environmental intelligence systems that support adaptive ecosystem management in an era of accelerating environmental change.

## 1 Introduction

As global climates become increasingly erratic, understanding plant responses to environmental stressors has become critically important for predicting ecosystem stability, agricultural productivity, and climate resilience (Ogwu, 2019; Ogwu, 2025; Ogwu and Izah, 2026). Stressors such as drought, herbivory, temperature extremes, nutrient limitations, and pollution trigger complex physiological and biochemical responses in plants. Many of these responses are rapidly communicated through the release of volatile organic compounds (VOCs), which function as highly dynamic chemical signals within and beyond plant tissues (Midzi et al., 2022; Pinto et al., 2010). These compounds function not only as internal metabolic regulators that influence plant defense and stress physiology, but also as external chemical cues that mediate interactions among plants, insects, microbes, and surrounding ecological communities (Kang et al., 2025). Consequently, real-time biosensing of VOC emissions offers a powerful, emerging approach for early detection of stress responses, enabling more adaptive, responsive management strategies in agriculture, forestry, and ecosystem monitoring systems (Luo et al., 2025).

The scientific understanding of plant VOCs has evolved substantially over the past decades. Initially regarded as passive metabolic by-products or secondary consequences of cellular processes, VOCs are now recognized as integral components of highly coordinated signaling networks that operate across multiple biological scales. Early experimental research demonstrated that mechanical damage and herbivore attack trigger highly specific and regulated VOC emission profiles that function directly in plant defense and communication (Werner et al., 2024). Compounds such as methyl jasmonate, for instance, play instrumental roles in activating defense signaling pathways and mediating interactions between plants and herbivores. Through such mechanisms, VOCs contribute to the formation of chemical communication networks that extend ecological interactions well beyond individual plants and immediate damage sites (Łabańska et al., 2022; Stoy et al., 2021).

Numerous studies demonstrate that plants exposed to VOCs emitted by stressed neighbors can alter their own metabolic activities, thereby priming defensive pathways and enhancing preparedness for anticipated stress conditions (De Cesare et al., 2025; Hu et al., 2009). Such inter-plant signaling processes extend the influence of VOCs from individual physiological responses to community-level coordination, suggesting that volatile emissions may function as distributed information systems within plant communities. In this sense, VOCs can serve as integrative indicators of plant community resilience and overall ecosystem health (Escobar-Bravo et al., 2024). Concurrently, advances in sensing technologies capable of detecting plant volatile emissions *in situ* have created new opportunities for continuous, non-invasive monitoring of plant health and environmental disturbances, significantly improving ecological forecasting, early warning systems, and adaptive ecosystem management (Frost et al., 2008).

This review is structured around the question of how plant VOCs can be understood as cross-scale stress indicators that connect intracellular stress responses with ecological communication, atmospheric filtering, and predictive monitoring systems. Conceptualizing plant VOCs as cross-scale indicators necessitates an integrative perspective that bridges molecular biology, plant physiology, ecological interactions, and ecosystem-level processes. VOC signals generated at the cellular and leaf levels can propagate outward, influencing neighboring plants, herbivore populations, natural enemies, and microbial communities, thereby shaping trophic dynamics and feedback processes across ecological networks (Frost et al., 2007). The ecological consequences of volatile emissions are therefore inherently context-dependent, varying according to species composition, environmental conditions, atmospheric chemistry, and the structural complexity of surrounding biological communities (Ederli et al., 2021; Peñaflor et al., 2011; Rasmann and Turlings, 2007). Understanding these multilayered and scale-bridging interactions is essential for translating plant volatile signaling from descriptive ecological phenomena into practical tools for environmental monitoring, risk assessment, and ecosystem management. Several recent reviews have examined plant VOC biosynthesis, inter-plant communication, or analytical and ecological dimensions of volatile signaling in relatively focused ways rather than within a single integrative framework (Ninkovic et al., 2020; Wang and Erb, 2022). In contrast, the present review synthesizes these strands within a unified cross-scale framework that conceptualizes plant VOCs as volatile stressometers linking molecular regulation, ecological communication, atmospheric transformation, and predictive monitoring technologies. In doing so, the review aims to show readers not only what plant VOCs do, but how their interpretation across scales can improve understanding of plant stress biology and support emerging applications in environmental monitoring and climate-resilient management.

This review examines current advances in plant VOC stress signaling across biological and ecological scales, emphasizing their role as volatile stressometers that translate physiological disturbances into measurable biochemical and ecological signals. Drawing on insights from molecular regulation, plant physiological processes, sensing technologies, ecological interactions, and emerging analytical frameworks, we demonstrate how stress-induced VOC emissions encode information about stress type, intensity, and temporal dynamics, enabling interpretation as dynamic indicators of plant health and environmental change. A unified cross-scale framework is developed in this review, connecting intracellular metabolic responses with community-level signaling and ecosystem-scale feedback processes through the integration of biochemical, ecological, technological, and applied perspectives. In this context, plant VOCs are not only products of stress responses but also functional mediators of plant–environment communication that influence neighboring organisms and broader ecological networks. Furthermore, their integration with emerging sensing platforms and analytical tools highlights their growing potential for precision agriculture, ecosystem monitoring, and predictive environmental management. In doing so, this review positions plant VOCs as central components of adaptive responses in changing ecosystems and as critical nodes linking molecular processes to landscape-scale resilience (Farag et al., 2013; Soriano and McCormick, 2020; Escobar‐Bravo et al., 2024).

## 2 Biochemical and molecular foundations of plant volatile stress responses

Plant VOCs function as volatile stressometers at the biochemical level, where stress-responsive metabolic pathways generate distinct emission signatures that encode information on the type, intensity, and temporal dynamics of environmental perturbations.

### 2.1 Major classes of plant volatile organic compounds

The major biochemical classes of plant VOCs differ in their biosynthetic origins, ecological functions, and stress-response signaling capacities. **Table 1** summarizes the dominant compound classes, their associated biosynthetic pathways, and the primary ecological roles linked to plant volatile stress signaling. Plant VOCs encompass a chemically diverse range of compounds that are central to defense regulation and ecological communication. The primary classes include terpenoids, green leaf volatiles (GLVs), benzenoids, and sulfur-containing compounds, each contributing differently to stress perception and signaling dynamics.

**Table 1:** Major classes of plant volatile organic compounds and their functional roles.

VOC class	Major biosynthetic pathway	Representative compounds	Primary ecological functions	Typical stress triggers
Terpenoids	MEP/Mevalonate pathways	Isoprene, monoterpenes, sesquiterpenes	Herbivore deterrence, predator attraction, atmospheric interactions	Heat, herbivory, drought
Green Leaf Volatiles (GLVs)	LOX-HPL pathway	Hexanal, hexenal, hexenol	Rapid wound signaling, plant–plant communication	Mechanical damage, herbivory
Benzenoids/Phenylpropanoids	Shikimate pathway	Methyl salicylate, benzaldehyde	Pollinator attraction, defense signaling	Pathogen infection
Sulfur-containing VOCs	Glucosinolate metabolism	Dimethyl sulfide	Herbivore deterrence, oxidative signaling	Biotic stress

VOC(s), Volatile Organic Compound(s); MEP, Methylerythritol Phosphate pathway; MVA, Mevalonic Acid pathway; LOX, Lipoxygenase; HPL, Hydroperoxide Lyase; LOX–HPL, Lipoxygenase–Hydroperoxide Lyase pathway; GLVs, Green Leaf Volatiles; DMS, Dimethyl Sulfide; MeSA, Methyl Salicylate.

Terpenoids represent the largest class of plant secondary metabolites and are characterized by remarkable structural diversity. They serve multiple ecological functions, including defense against herbivores, attraction of pollinators, and contribution to plant aromatic profiles (Engelberth, 2020; Kazimírová et al., 2021). In stress contexts, terpenoids may function as both direct defensive agents and indirect signaling molecules that recruit natural enemies of herbivores, thereby extending their influence beyond the emitting plant. GLVs are primarily emitted following leaf wounding or herbivory and consist largely of short-chain aldehydes, alcohols, and esters such as 1-hexanal (Engelberth, 2020; Shiojiri et al., 2006). These compounds are among the most rapidly produced stress volatiles and function as early-warning signals that inform neighboring plants of impending threats. Various studies have demonstrated that GLV emissions act not only as direct deterrents to herbivores but also as prime defense responses in nearby plants, thereby strengthening collective resistance within plant communities (Kutty and Mishra, 2023; Das et al., 2025). Benzenoids, derived from the shikimic acid pathway, are particularly notable for their roles in floral scent production and pollinator interactions. However, they also participate in defense-related signaling processes under stress conditions (Mostafa et al., 2022a). Their aromatic properties and metabolic regulation enable them to operate at the interface between ecological attraction and stress-responsive communication. Sulfur-containing compounds, including those derived from glucosinolate metabolism, represent another important class of plant VOCs involved in stress modulation (Ngumbi et al., 2022; Tanaka et al., 2021). These compounds can deter herbivores while also participating in signaling cascades associated with cellular responses to both biotic and abiotic stressors (Ngumbi et al., 2022). Their production often reflects tightly regulated metabolic shifts triggered by environmental perturbations.

### 2.2 Biosynthetic pathways and metabolic regulation

The biosynthesis of VOCs involves complex pathways regulated by multiple enzymes, signaling networks, and metabolic controls. Although the principal biochemical routes underlying plant VOC biosynthesis—including the lipoxygenase (LOX), methylerythritol phosphate (MEP), mevalonate, and shikimate pathways—are broadly conserved across many plant taxa, the resulting volatile phenotypes are far from uniform. The composition, abundance, inducibility, and ecological deployment of emitted VOC blends vary substantially among species, lineages, tissues, developmental stages, and environmental contexts, reflecting differences in gene regulation, metabolic flux, stress sensitivity, and ecological function (Laothawornkitkul et al., 2009; Dudareva et al., 2013; Picazo-Aragonés et al., 2020). Thus, conservation at the level of core biosynthetic architecture does not imply uniformity in volatile output, because downstream regulation and pathway crosstalk generate highly variable emission profiles across taxa. For instance, the lipoxygenase (LOX) pathway is paramount in the production of green leaf volatiles (GLVs), where the enzyme catalyzes the oxidation of polyunsaturated fatty acids, generating C6 aldehydes and alcohols (Vincenti et al., 2019; Hashem et al., 2022). Specifically, the action of lipoxygenases is followed by hydroperoxide lyases (HPLs), which cleave hydroperoxide fatty acids into volatile intermediates (Kazimírová et al., 2021; Qian et al., 2016). In parallel, terpenoid biosynthesis is supported by the plastidial MEP pathway and the cytosolic mevalonate pathway, which are themselves interconnected through metabolic crosstalk and contribute to the diversity of isoprene, monoterpene, and sesquiterpene emissions (Dudareva et al., 2013; Picazo-Aragonés et al., 2020). Benzenoid and phenylpropanoid volatiles are primarily derived from the shikimate pathway and its downstream branches, further adding to the complexity and specificity of plant volatile blends (Laothawornkitkul et al., 2009; Picazo-Aragonés et al., 2020).

Recent advancements in bioengineering have allowed for the optimization of these enzymes to enhance GLV production for applications in food flavoring and agriculture (Vincenti et al., 2019; Gigot et al., 2012). At the same time, the regulation of these biosynthetic genes is intricate and highly context-dependent, often mediated by environmental signals, developmental cues, and stress-responsive hormonal pathways, leading to substantial variations in VOC emission profiles under different stress conditions (Picazo-Aragonés et al., 2020; Kanjana et al., 2026; Laothawornkitkul et al., 2009). This regulatory plasticity is central to the role of VOCs as stress-responsive outputs, because it enables plants to generate volatile blends that are not only chemically diverse but also ecologically tuned to specific biotic and abiotic challenges.

### 2.3 Stress-responsive transcriptional and signaling networks

Upon encountering stress, plants activate complex transcriptional networks that orchestrate the biosynthesis of VOCs. The signaling molecule jasmonate plays a central role in this process, and studies indicate that the interaction between jasmonate signaling and other hormones (e.g., salicylic acid) is crucial for regulating the defensive response (Ngumbi et al., 2022; Qi et al., 2011). Hormonal shifts triggered by stress can alter the expression of LOX and HPL genes, highlighting a multifaceted regulatory mechanism (Maynard et al., 2021; Hu et al., 2023). The integration of signals from biotic stressors (e.g., herbivores) and abiotic factors (e.g., drought) can potentiate the transcriptional activation of defense-related genes, modulating not just VOC emissions, but also broader metabolic pathways (Tong et al., 2012; Gorman et al., 2021). Understanding these networks is vital for developing strategies to enhance crop resilience against pests and diseases (Shiojiri et al., 2006; Hamid et al., 2023).

### 2.4 Hormonal regulation: JAs, salicylates, ethylene, and ABA pathways

Hormonal regulation within plants significantly influences VOC production. Jasmonic acid and its methyl ester are key hormones in the defense response, prompting the biosynthesis of terpenoid and benzenoid compounds (Wang et al., 2020; Qian et al., 2016). Ethylene is also implicated in promoting the emission of stress-induced VOCs, particularly during plant reproductive stages (Mostafa et al., 2022b). The interplay between these hormonal pathways creates a dynamic stress response, allowing plants to optimize energy use toward defensive strategies while balancing growth (Qi et al., 2011; Das et al., 2025). Additionally, abscisic acid (ABA), primarily known for its role in drought response, has been shown to participate in VOC signaling pathways, where it can either promote or inhibit volatile emissions depending on the context (Palai et al., 2023; Gorman et al., 2021).

**Figure 1:**
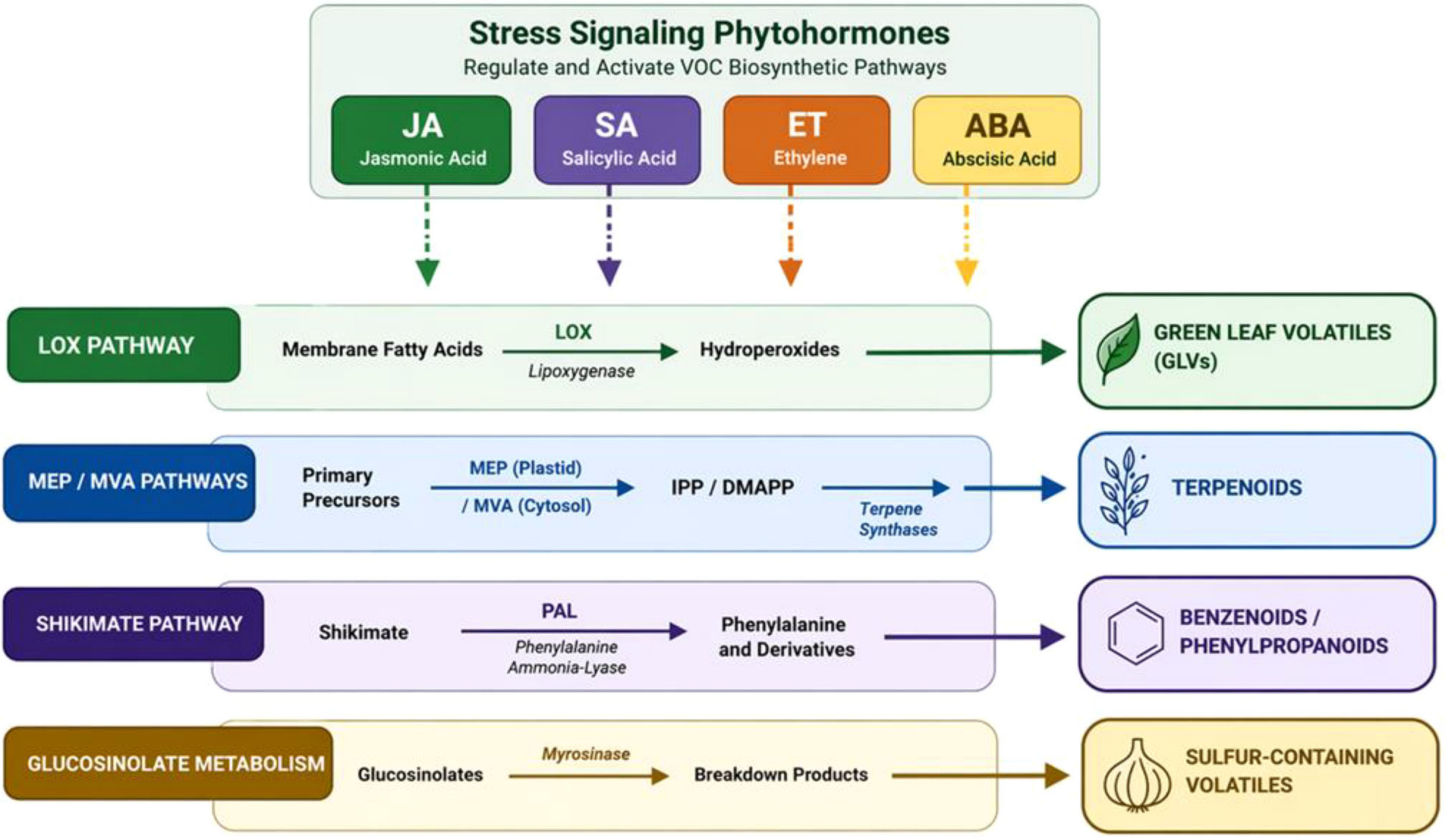
Biochemical pathways of stress-induced volatile organic compound production in plants. JA, Jasmonic Acid; SA, Salicylic Acid; ET, Ethylene; ABA, Abscisic Acid; LOX, Lipoxygenase; MEP, Methylerythritol Phosphate pathway (plastidial); MVA, Mevalonate pathway (cytosolic); PAL, Phenylalanine Ammonia-Lyase; GLVs, Green Leaf Volatiles; Dashed arrows – Hormonal regulation (activation/modulation); Solid arrows – Biosynthetic pathway/metabolic flow.

Major plant stress-responsive metabolic pathways responsible for the biosynthesis of VOCs include the lipoxygenase (LOX) pathway, which produces green leaf volatiles (GLVs). In contrast, the plastidial methylerythritol phosphate (MEP) pathway and the cytosolic mevalonate pathway generate terpenoid VOCs (**Figure 1**). The shikimate pathway leads to benzenoid and phenylpropanoid compounds, and glucosinolate metabolism produces sulfur-containing volatiles. Stress signaling phytohormones—including jasmonic acid (JA), salicylic acid (SA), ethylene (ET), and abscisic acid (ABA)—regulate the activation and intensity of these pathways, coordinating plant defensive and adaptive responses to biotic and abiotic stress conditions.

### 2.5 Genetic variability and species-specific volatile signatures

The variability in VOC profiles across different plant species can be substantial and is often determined by genetic factors. Studies have shown that different genetic backgrounds influence the expression of the LOX-HPL pathway, leading to distinct volatile signatures associated with specific stressors (Engelberth, 2020; Qian et al., 2016). For example, different grape varieties exhibit divergent patterns of GLV emission, which can influence both flavor profiles and plant interactions with herbivores and pollinators (Engelberth, 2020; Hamid et al., 2023). Furthermore, genetic studies using transgenic approaches have demonstrated that manipulating the expression of key biosynthetic genes can enhance VOC production, thereby increasing resistance to herbivores and pathogens (Fits and Memelink, 2000; Qi et al., 2024). This genetic variability opens avenues for breeding programs to enhance stress resilience by selecting for specific volatile profiles (Tian et al., 2018; Picazo-Aragonés et al., 2020; Kanjana et al., 2026).

## 3 Abiotic stress drivers of volatile emissions

Abiotic stressors drive plant VOC emissions, positioning them as volatile stressometers of environmental perturbation. Distinct stress conditions, such as heat, drought, salinity, nutrient limitation, and atmospheric pollution, generate characteristic emission profiles that encode the intensity, duration, and combinatorial nature of physiological stress responses.

### 3.1 Heat and temperature extremes

Heat stress and temperature extremes significantly impact volatile emissions from plants, influencing their physiological and biochemical processes. As temperatures exceed optimal levels, plants experience elevated metabolic rates, leading to increased synthesis of VOCs that are essential for signaling stress responses and attracting beneficial organisms. Munné-Bosch et al. (2004) noted that high-temperature exposure compromised the antioxidant protective mechanisms in plants such as Holm Oak, ultimately affecting their growth and reproductive success under drought conditions. Moreover, Khalid et al. (2025) discussed how extremes can disrupt not only growth but also critical developmental processes, such as flower and fruit formation, thereby negatively influencing overall yield.

### 3.2 Drought and water stress

Drought is a principal abiotic stress affecting crop performance worldwide, resulting in substantial yield losses. As stressed plants activate various phytohormone-mediated signaling pathways, they also enhance the biosynthesis of secondary metabolites and volatile compounds that help defend against oxidative damage (Vwioko et al., 2018; Ogwu, 2025). Jogawat et al. (2021) provided insights into the systemic activations within the plant that facilitate drought tolerance, linking the production of stress-related VOCs with intra-plant communication and resilience. The interplay of stress signaling cascades also integrates responses to stomatal closure, a key mechanism for conserving water during periods of low moisture (Xu et al., 2024).

### 3.3 Salinity stress

Salinity poses a significant challenge to plant growth, especially in arid regions where soil salinization commonly occurs due to poor irrigation practices. Chaudhry et al. (2022) indicated that salt stress disrupts ionic balance and osmotic potential in plants, initiating adaptive responses that include the release of stress-related volatiles. These VOCs play a dual role, modulating plant-plant interactions under stress conditions and signaling to neighboring plants. Additionally, the production of compatible solutes, such as proline, which accumulates under salinity stress, has been closely associated with enhanced plant resilience (Rani and Tokas, 2020). Furthermore, arbuscular mycorrhizal fungi can ameliorate the negative impacts of salinity through enhanced nutrient uptake, which correlates with improved emission profiles of beneficial volatiles (Wahab et al., 2023; Aggarwal et al., 2012).

### 3.4 Nutrient limitation and soil degradation

The health of soil directly influences plant growth and stress-related emissions. Várallyay emphasizes how nutrient deficiencies and soil degradation processes can lead to poor plant health, consequently impairing their ability to respond to abiotic stress (Várallyay, 2002). Nutrient availability affects plant metabolic pathways, which, in turn, determine the levels of stress-signaling-associated VOCs emitted. Hu et al. (2010) reviewed the interplay among multifunctional genes involved in stress resistance, noting that nutrient uptake and effective signaling are critical for plant response robustness. The effects of these deficiencies are compounded in degraded soils, leading to diminished plant resilience and reduced volatile production.

### 3.5 Atmospheric pollution and oxidative stress

Atmospheric pollutants exacerbate stress responses and affect the VOC emission profiles of plants. Munné-Bosch et al. (2004) linked air impurities to oxidative stress and alterations in phytohormonal dynamics, which impact photosynthesis and metabolic efficiency. Knieper et al. (2023) indicated that reactive oxygen species (ROS) act as key signaling molecules under stress conditions, promoting the production of defensive compounds while also necessitating precise regulatory mechanisms to prevent cellular damage. The VOCs emitted in response to oxidative stress can also serve as signals to attract natural enemies of herbivores or as markers to initiate defense pathways in neighboring plants.

### 3.6 Combined and cascading stress effects

The simultaneous impacts of multiple abiotic stressors often lead to cascading effects that complicate plant responses. Drought, salinity, and temperature extremes frequently co-occur, creating a complex environment that strains plant adaptive capacity. Localized studies, such as those by Tamang and Fukao, demonstrate that plants exhibit integrated responses to multiple stresses, which include heightened VOC emissions that serve as indicators of physiological status and stress levels (Tamang and Fukao, 2015). Moreover, the phenomenon of cross-tolerance, in which prior exposure to one form of stress enhances resistance to others, underscores the importance of understanding these interconnected stress responses, as emphasized by Cortleven et al. (2019) in the context of phytohormones such as cytokinins.

## 4 Biotic stress and ecological defense signaling

At the ecological scale, plant VOCs function as volatile stressometers, propagating stress information across organisms and mediating interactions among neighboring plants, herbivores, and microbial communities through context-dependent signaling networks.

### 4.1 Herbivore-induced plant volatiles

Herbivore-induced plant volatiles (HIPVs) are a critical component of plant defense strategies, serving both to deter herbivores and to recruit natural enemies. When attacked by herbivores, plants emit specific VOCs that can serve as signals to other plants and organisms in the ecosystem. These HIPVs can vary significantly in composition depending on the type of herbivore and the extent of damage (Kumar et al., 2017; Zheng et al., 2025). For instance, studies have shown that plants such as *Arabidopsis thaliana* exhibit distinct volatile profiles when infested by different herbivores, thereby facilitating the attraction of specific predators and parasitoids (Kumar et al., 2017; Stirling et al., 2024). Additionally, the timing and quantity of HIPV emissions can be fine-tuned based on immediate pest presence, showcasing an adaptive signaling mechanism (Kutty and Mishra, 2023).

### 4.2 Pathogen-induced volatile signaling

Pathogen attacks also trigger robust volatile signaling in plants, activating metabolic pathways that enhance systemic defenses. The compounds released during pathogen infection not only help ward off pathogens but also induce defensive responses in neighboring plants (Howard et al., 2021). This phenomenon implicates VOCs as integral players in plant-to-plant communication, where signals can modulate the neighboring plant’s biochemical makeup in preparation for potential infections. The complexity of these interactions suggests an evolutionary advantage, as plants exhibiting heightened defensive states can better withstand subsequent attacks (Howard et al., 2021; Raza et al., 2021). For example, certain pathogens provoke a rapid release of volatile compounds such as green leaf volatiles, which can enhance the defensive capabilities of nearby plant communities through a cascading network of chemical communication (Kutty and Mishra, 2023).

### 4.3 Plant–microbe signaling interactions

The interactions between plants and their associated microbial communities in the rhizosphere represent another intricate layer of ecological communication. Microbial volatile organic compounds (mVOCs), meaning low-molecular-weight volatile compounds released by bacteria and fungi, can significantly influence plant health and growth by modifying plant metabolic pathways and promoting nutrient uptake. mVOCs are secreted by beneficial microbes and can significantly influence plant health and growth by modulating plant metabolic pathways and promoting nutrient uptake (Raza et al., 2021). Research indicates that mVOCs play dual roles: they mediate allelopathic interactions between plants and suppress pathogenic responses, while also enhancing plant growth-promoting activities (Vasseur-Coronado et al., 2021; Jain et al., 2024). Microbial species such as *Bacillus* tend to release specific mVOCs that can directly promote plant growth, indicating that the rhizosphere is a dynamic arena for volatile-mediated interactions influencing plant resilience and overall ecosystem health (Jain et al., 2024). For example, bacterial mVOCs such as 2,3-butanediol and acetoin produced by *Bacillus* species have been shown to stimulate plant growth and induce systemic resistance. Similarly, fungal volatiles such as 6-pentyl-α-pyrone emitted by *Trichoderma* spp. can enhance root development and activate defense responses. Other compounds, including dimethyl disulfide (DMDS) and indole, contribute to nutrient mobilization, pathogen suppression, and signaling processes that regulate plant–microbe and plant–plant interactions.

### 4.4 Multi-trophic ecological communication networks

The ecological networks established through volatile communication extend beyond direct plant-microbe interactions to encompass broader multi-trophic levels involving herbivores and their natural enemies. For instance, the reception of HIPVs can trigger responses in predators, creating a triadic ecological relationship that enhances plants’ defenses against herbivores while facilitating predator attraction (Kumar et al., 2017; Sharifi and Ryu, 2020). Such networks illustrate how a breakdown at one point—whether due to an abiotic stressor or a novel pest invasion—can disrupt the entire ecological balance and affect plant productivity, underscoring the need for integrated pest management strategies that account for these complex interactions (Sharifi and Ryu, 2020; Stirling et al., 2024).

**Figure 2:**
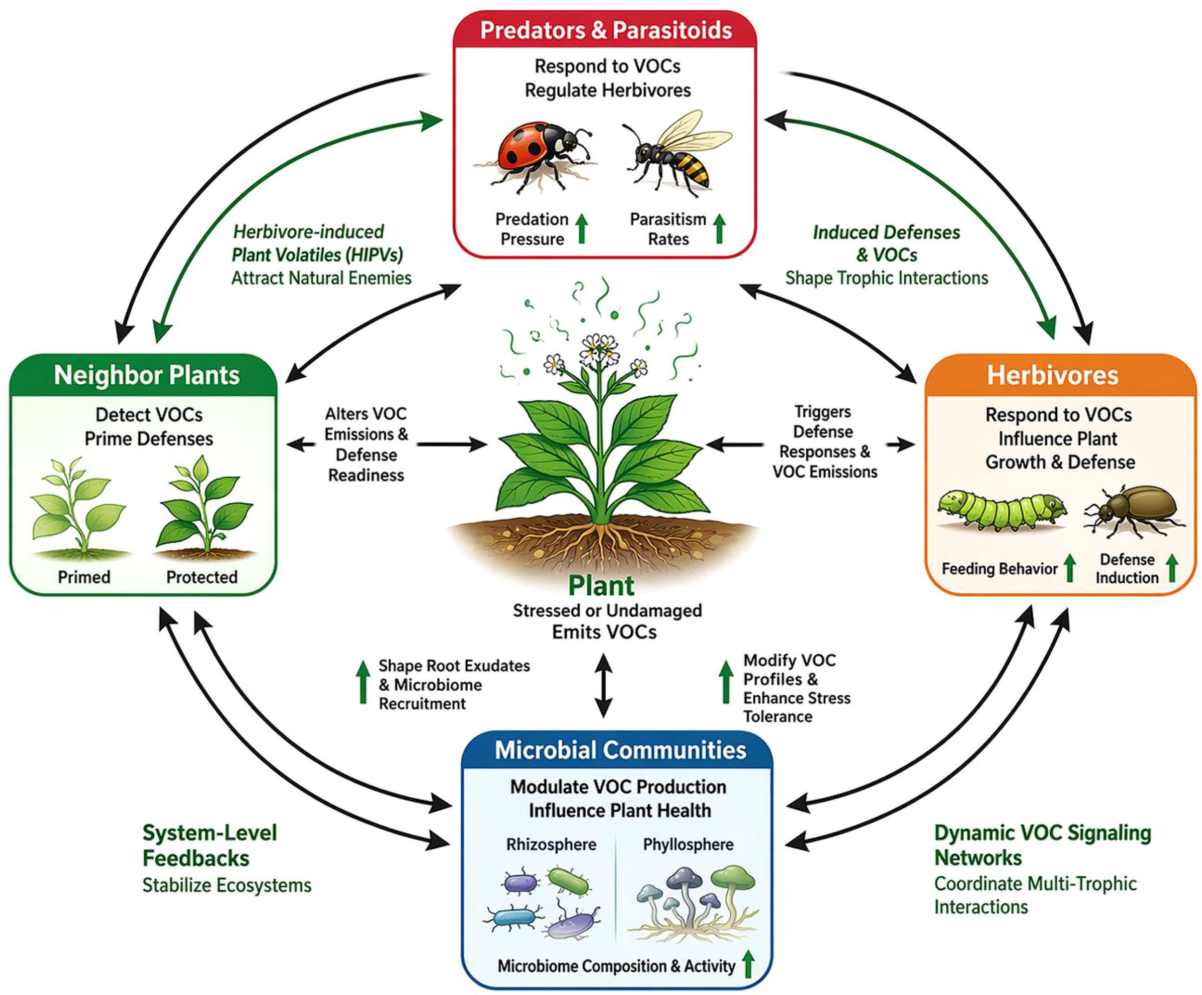
Conceptual multi-trophic ecological communication network mediated by plant volatile organic compounds.

**Figure 2** illustrates a conceptual multi-trophic ecological communication system mediated by plant VOCs. Stress-induced VOC emissions from plants influence neighboring plants, herbivores, predators, and parasitoids, as well as soil and phyllosphere microbial communities, creating interconnected signaling pathways across trophic levels. Bidirectional feedback loops indicate how these interacting organisms can both respond to and modify plant volatile emissions, thereby shaping plant defense strategies, trophic interactions, and ecosystem stability. **Figure 2** highlights the complexity of VOC-mediated ecological signaling as a dynamic, feedback-regulated communication network spanning plant, insect, and microbial communities.

### 4.5 Mechanisms of volatile perception in receiving organisms

A central mechanistic question in volatile stress biology is how emitted compounds are perceived by receiving organisms and translated into downstream physiological or behavioral responses. In neighboring plants, volatile perception is increasingly understood as a multi-step process involving uptake, signal conversion, and integration into canonical defense and stress-response networks. Volatile entry may occur through stomata, cuticular surfaces, or diffusion across cellular interfaces, after which compounds or their derivatives can alter membrane properties, cellular redox status, or intracellular signaling states (Wang and Erb, 2022; Aguirre et al., 2023). Emerging evidence further suggests that volatile exposure can activate early signaling events such as calcium influx, reactive oxygen species accumulation, and mitogen-activated protein kinase cascades, which subsequently intersect with jasmonic acid, salicylic acid, ethylene, and abscisic acid pathways to regulate defense priming and stress acclimation (Wang and Erb, 2022). For green leaf volatiles and other herbivore-induced cues, these responses are often associated with priming rather than immediate full defense activation, allowing receiver plants to mount faster or stronger responses upon subsequent challenge. At the same time, dedicated high-affinity receptors for many plant VOCs remain incompletely resolved, indicating that volatile perception in plants may rely on a combination of physicochemical uptake, transport processes, metabolic conversion, and signal integration rather than a single universal receptor system (Wang and Erb, 2022).

In insects, volatile perception is more clearly characterized and depends on specialized olfactory systems located primarily in antennal sensilla. Plant volatiles enter through pores in the sensillar cuticle and are transported through receptor lymph by odorant-binding proteins and chemosensory proteins to olfactory receptor neurons, where ligand-selective odorant receptors and co-receptors convert chemical signals into neural activity that drives host recognition, oviposition decisions, avoidance behavior, and attraction to herbivore-induced plant cues (Qi et al., 2024; Zhang et al., 2023). These mechanisms are especially important in herbivores, parasitoids, and predators that exploit plant-emitted volatiles to locate hosts, prey, or suitable feeding and reproductive environments.

Volatile perception in microbial systems remains less well resolved. However, current evidence indicates that microbial volatile compounds can alter membrane properties, redox balance, transcriptional regulation, and hormone-associated signaling in plants, while also reshaping microbial behavior in the rhizosphere (Sharifi and Ryu, 2018). Such responses can influence microbial growth, motility, antagonistic activity, colonization, and biofilm formation, thereby extending the significance of volatile signaling beyond plant–plant communication to broader plant–microbe interaction networks. This unresolved but rapidly developing area remains important because the utility of VOCs as stress indicators depends not only on what plants emit but also on how reliably these signals are taken up, decoded, and translated into biological responses by neighboring plants and associated organisms.

### 4.6 Evolutionary ecology of volatile-mediated defense

The evolutionary implications of volatile-mediated communication suggest a continuous co-evolutionary dynamic between plants and their microbial associates. Plants have been shown to adapt their volatile emissions in response to varying environmental conditions and biotic pressures, leading to the development of specialized microbial communities in the rhizosphere that enhance their ability to cope with stressors (Sarsaiya et al., 2025). These interactions highlight the delicate balance between fitness costs and benefits among plant and microbial partners, in which plants may evolve to emit distinct volatile profiles that favor beneficial microbes while deterring pathogens (Klein et al., 2022). Understanding these aligned evolutionary pathways can provide key insights into optimizing sustainable agricultural practices and managing crop health (Sarsaiya et al., 2025; Bohm et al., 2017).

**Figure 3:**
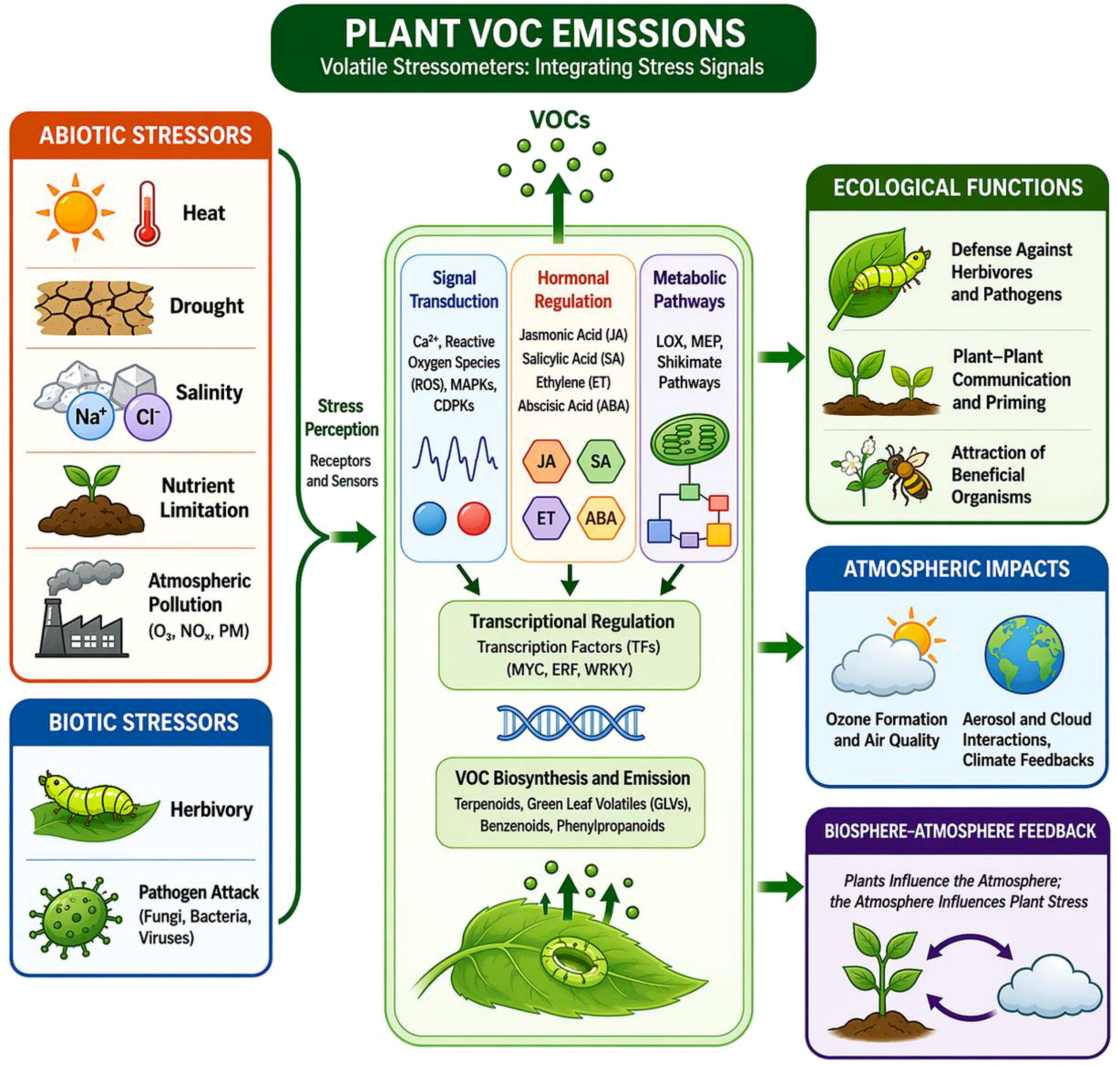
Abiotic and biotic stress drivers converging on plant VOC emissions as volatile stressometers, showing how diverse environmental and biological stressors—including heat, drought, salinity, nutrient limitation, herbivory, pathogen attack, and atmospheric pollution—activate interconnected physiological, hormonal, and metabolic pathways that regulate VOC release.

Plants experience a wide range of abiotic and biotic stressors, including heat stress, drought, herbivory, pathogen attack, and environmental pollution, which activate complex physiological and biochemical response pathways. A key outcome of these responses is the emission of VOCs, which function as integrative signals linking stress perception to ecological and atmospheric processes. **Figure 3** provides an overview of this broader ecological logic by showing how multiple abiotic and biotic stressors converge on VOC emission pathways, positioning plant volatiles as integrative stress signals that connect physiological stress perception with ecological interactions and biosphere–atmosphere feedbacks. These converging stress inputs shape volatile outputs that influence plant defense against herbivores and pathogens, intra- and inter-plant communication, and atmospheric processes relevant to air quality and climate interactions. In this sense, VOC emissions function not merely as by-products of stress, but as central mediators through which plants translate environmental disturbance into ecologically and atmospherically consequential information streams.

Plant VOC emission patterns are shaped by interacting abiotic and biotic stressors that activate distinct metabolic pathways and signaling networks. **Table 2** summarizes key stress drivers and their associated volatile signaling responses.

**Table 2:** Abiotic and biotic drivers of stress-induced VOC emissions.

Stress category	Specific stressor	Primary physiological response	Typical VOC response	Ecological function
Abiotic	Heat stress	Membrane destabilization and ROS production	Increased isoprene emissions	Thermal protection and atmospheric feedback
Abiotic	Drought	Stomatal closure and ABA activation	Terpenoid and GLV shifts	Stress priming and plant communication
Abiotic	Salinity	Ionic imbalance	Stress-related secondary volatiles	Defense signaling
Biotic	Herbivory	Jasmonate activation	HIPVs (terpenoids and GLVs)	Predator recruitment
Biotic	Pathogens	SA signaling	Benzenoid compounds	Induced systemic resistance

ROS, Reactive Oxygen Species; ABA, Abscisic Acid; GLVs, Green Leaf Volatiles; HIPVs, Herbivore-Induced Plant Volatiles; SA, Salicylic Acid.

## 5 Integration across biological scales: from cells to ecosystems

The integration of plant volatile stress signals across multiple biological scales is essential for understanding how plants respond to environmental changes and communicate within and between species at the cellular, whole-plant, community, landscape, and ecosystem levels. Plant volatile stress signals operate across nested biological and ecological scales, linking intracellular processes to ecosystem-level feedbacks. **Table 3** summarizes the multi-scale structure of volatile signaling systems.

**Table 3:** Cross-scale integration of plant volatile signaling.

Biological scale	Key processes	VOC role	Monitoring potential
Cellular	Gene expression, metabolic activation	Intracellular signaling molecules	Metabolomics
Whole-plant	Physiological stress responses	Internal and external signaling	Proximal sensing
Community	Plant–plant communication	Defense priming, ecological signaling	Field sensors
Landscape	VOC flux interactions	Atmospheric chemistry interactions	Unmanned Aerial Vehicle/satellite sensing
Ecosystem	Climate feedbacks	Aerosol formation, ecosystem indicators	Earth-system monitoring

Plant VOCs should be viewed as one major component of plant stress integration rather than the sole signaling mechanism linking molecular responses to ecological and ecosystem outcomes. Alongside VOCs, plants rely on additional signaling modalities, including mobile non-volatile metabolites, electrical signals, hydraulic signals, and systemic hormonal transport, all of which contribute to stress perception, signal amplification, and long-distance coordination. Mechanistic evidence for VOC function is strongest at the molecular, cellular, and organismal levels, where biosynthesis, emission, uptake, perception, and downstream signaling have been increasingly resolved (Wang and Erb, 2022). By contrast, scaling these processes to landscape- and ecosystem-level dynamics currently depends on hierarchical ecological interpretation, atmospheric transport and degradation processes, and coupled biophysical models rather than a single universally validated mechanistic framework (Holopainen and Blande, 2013; Blande et al., 2014).

### 5.1 Cellular-level metabolic signaling integration

At the cellular level, plants have evolved complex biochemical pathways to respond to abiotic and biotic stresses. The perception of stress signals triggers defense mechanisms that operate primarily in the apoplast, the space outside plant cells, accumulating molecules such as ascorbate and secondary metabolites before the cellular processes are significantly affected (Du et al., 2024). The synthesis of various secondary metabolites not only serves defensive roles but also contributes to a metabolic network that can adapt to environmental stresses (Midzi et al., 2022; Isah, 2019). Recent investigations underscore how metabolites function as signaling molecules, integrating metabolic responses at the cellular level with environmental stimuli while minimizing metabolic costs (Du et al., 2024).

Moreover, VOCs act as critical signals that mediate intra- and intercellular communication, preparing neighboring cells for potential stressors even before they arise. This phenomenon exemplifies anticipatory responses in plants, highlighting the predictive power of plant signaling mechanisms (Midzi et al., 2022; Ninkovic et al., 2020). The diverse profiles of VOCs emitted under stress conditions thus reflect complex regulatory networks within plant cells, showcasing the intricate relationships between ecological context and physiological status.

### 5.2 Whole-plant physiological responses

At the whole-plant level, physiological responses to stress involve alterations in growth and metabolic pathways. The adaptive strategies employed by plants under drought or salinity conditions can lead to significant shifts in volatile emissions, affecting not only plant health but also community dynamics (Midgley, 2017; Midzi et al., 2022). The adjustment of these emissions can prime other plants, mitigating shared risks (Midzi et al., 2022; Ninkovic et al., 2020). For instance, some plants are capable of “eavesdropping” on stress-induced signals from neighboring plants, allowing them to preemptively activate their defense mechanisms without direct exposure to the stressor (Midzi et al., 2022; Ninkovic et al., 2020).

Recent studies also examine the roles of essential nutrients like potassium, which influences various physiological processes linked to stress responses (Sardans and Peñuelas, 2021). Drought stress has been shown to trigger specific pathways that upregulate the expression of genes involved in stress tolerance, leading to enhanced production of osmoprotectants and antioxidants, which help maintain cellular integrity (Mathivanan, 2021; Aslam et al., 2022). These physiological adaptations at the whole-plant level are crucial for optimizing plant fitness in increasingly variable environments.

### 5.3 Community-level signaling and plant–plant communication

At the community level, plant interactions facilitated by VOCs are critical for maintaining ecological balance. These volatiles serve as “signals” that communicate stress conditions to neighboring plants, influencing their physiological states and enhancing community resilience. For instance, research illustrates how some species can emit protective volatiles in response to herbivore damage, effectively warning neighboring plants of potential threats (Ninkovic et al., 2020; Cofer et al., 2018). The networking of volatile signals in plant communities demonstrates the ecological specificity and context-dependent nature of plant interactions. Environmental factors, including pollution and varying climates, can alter these signaling pathways, thereby altering community dynamics (Blande et al., 2014). Additionally, plants may develop varying degrees of tolerance depending on their neighboring flora and the prevalence of beneficial microorganisms in their rhizospheres (Chieb and Gachomo, 2023; Portal-González et al., 2025). This mutualistic relationship underscores the need to examine plant interactions holistically, considering both above- and below-ground dynamics.

### 5.4 Landscape-scale volatile fluxes

At the landscape scale, VOC fluxes significantly impact regional ecosystems and atmospheric processes. Studies suggest that as climate conditions, such as temperature and humidity, change, they can alter the rates of VOC emissions, thereby affecting air quality and cloud chemistry (Wang et al., 2018; Prugh et al., 2018). For example, elevated temperatures can increase isoprene emissions, which are important for cloud formation and have implications for local climate regulation (Durand et al., 2025; Bacheva et al., 2025). Furthermore, models indicate that these landscape-level interactions involve not only the direct physiological responses of plants but also feedback loops that include community composition and ecosystem productivity (Shugart et al., 2020; Midzi et al., 2022). Notably, certain plant species may respond differently to temperature changes, revealing the complexity of species interactions at the landscape level and their implications for biodiversity conservation and ecosystem management (Prugh et al., 2018; Bacheva et al., 2025). Although VOC flux measurements are increasingly incorporated into atmospheric and Earth-system models, direct empirical datasets linking plant VOC fluxes to ecosystem resilience, productivity, or other integrated ecological performance metrics remain comparatively limited. At present, the strongest large-scale linkages are to atmospheric chemistry, aerosol formation, and climate feedback processes rather than to fully resolved ecosystem function indicators such as resilience or productivity (Laothawornkitkul et al., 2009).

### 5.5 Ecosystem feedbacks and climate interactions

At the ecosystem level, the feedback mechanisms resulting from plant volatile emissions play a crucial role in shaping ecological outcomes under climate change. As plants emit VOCs, these compounds can influence not only local microclimates but also larger-scale climatic processes, such as atmospheric chemistry and aerosol formation, which, in turn, affect plant performance and community structure (Wang et al., 2018; Prugh et al., 2018). Moreover, interactions between abiotic stresses like drought and heat and their combined effects on volatile emissions highlight the interconnectedness of climate events and plant responses (Cofer et al., 2018; Papazian et al., 2016). Understanding how these multiple stressors interact to influence plant communities and their resilience is crucial for predicting future ecosystem dynamics and for implementing conservation strategies to bolster ecosystem health and sustainability in the face of climate change (Mathivanan, 2021; Portal-González et al., 2025). Enhanced knowledge in these areas is essential for developing resilient agricultural practices and promoting biodiversity conservation in changing environmental contexts.

**Figure 4:**
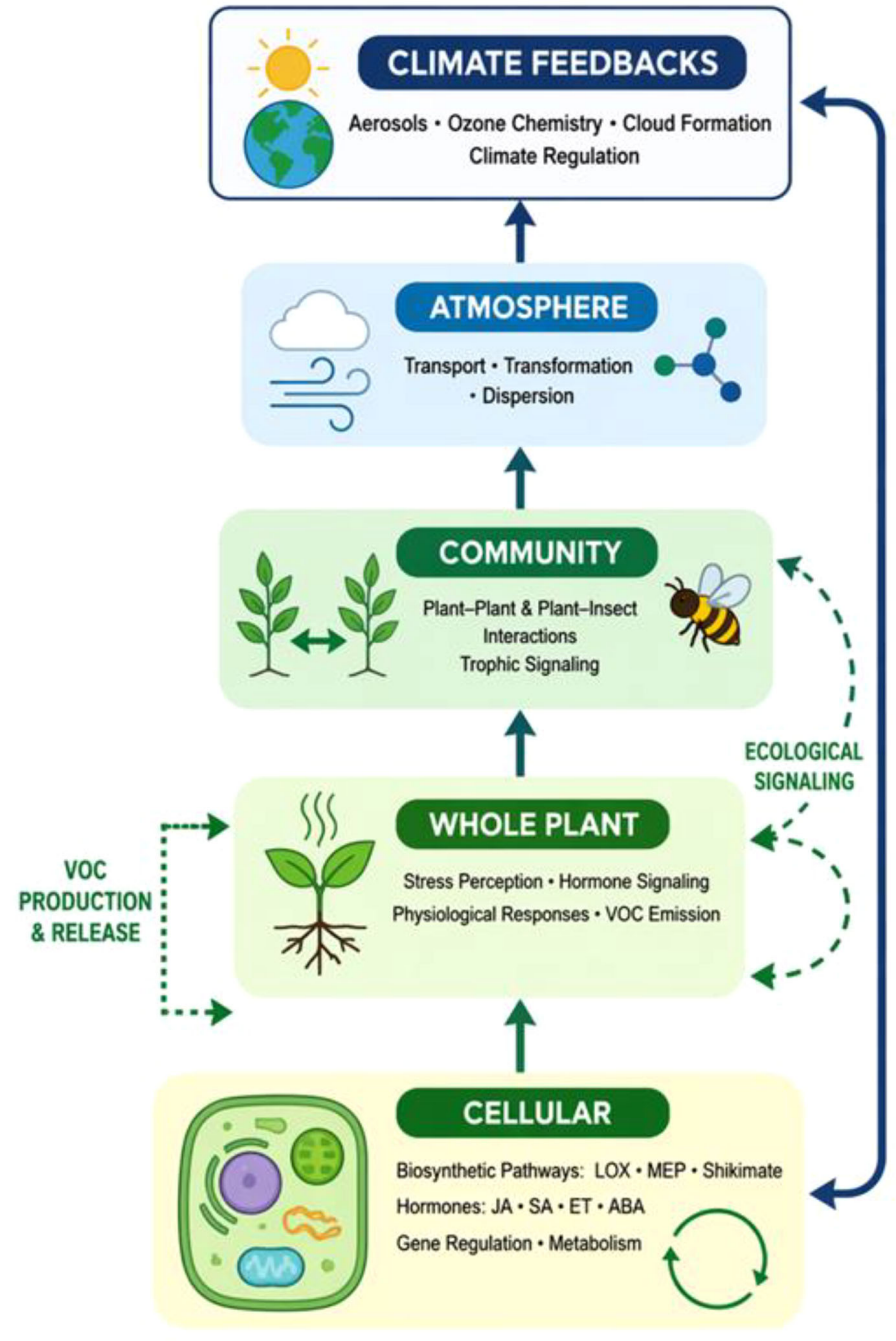
Cross-scale architecture of plant VOCs as volatile stressometers, illustrating how stress-induced biochemical and physiological responses are translated across biological and ecological scales—from cellular signaling and whole-plant responses to community communication, atmospheric transport, and ecosystem-level feedbacks.

Rather than operating as isolated biochemical outputs, these volatile signals connect intracellular metabolic regulation with broader ecological and atmospheric dynamics, illustrating how stress information is generated, transmitted, and amplified across biological scales (**Figure 4**). Beginning at the cellular and tissue levels, stress-induced biochemical processes drive VOC synthesis and release, which subsequently propagate through whole-plant physiological responses, plant–plant and plant–insect communication at the community level, landscape-scale atmospheric dispersion, and broader climate feedback interactions. It highlights how localized biochemical signaling mechanisms scale upward to influence ecosystem interactions and atmospheric processes, establishing plant volatile signaling as a critical cross-scale communication system linking cellular stress responses to global ecological and climate dynamics.

## 6 Technologies for detecting and quantifying plant volatile stress signals

The study of plant VOCs, particularly under stress conditions, has gained substantial attention due to their critical role in plant communication, pest deterrence, and responses to environmental changes. Technologies available for detecting and quantifying these signals may be subdivided into approaches ranging from laboratory platforms to advanced field-deployable technologies. Advances in sensing and analytical technologies enable plant VOCs to be captured and decoded as quantifiable volatile stressometers, transforming transient emission signals into high-resolution data streams for real-time assessment of plant stress and environmental conditions.

### 6.1 Laboratory analytical platforms

Laboratory analytical platforms play a pivotal role in the identification and quantification of plant VOCs. Among the most prevalent techniques are Gas Chromatography-Mass Spectrometry (GC-MS), Proton Transfer Reaction Mass Spectrometry (PTR-MS), and Selected Ion Flow Tube Mass Spectrometry (SIFT-MS).

1. Gas Chromatography-Mass Spectrometry (GC-MS) is regarded as one of the most reliable and established methods for analyzing complex mixtures of VOCs. It enables detailed chemical profiling, making it especially valuable for metabolic studies and stress response analyses (Cuaran and León, 2021; Pham and Beauchamp, 2022). GC-MS enables effective separation and quantification of volatile compounds derived from plant tissues, thereby contributing significantly to genetic and biochemical research (Frolova, 2025).
2. Proton Transfer Reaction Mass Spectrometry (PTR-MS) offers several advantages over traditional methods, including the ability to perform real-time analysis of VOCs with minimal sample preparation. This technique is particularly beneficial for studying dynamic changes in VOC emissions from plants under stress conditions, as it provides rapid feedback necessary for ecological monitoring (Davison et al., 2008; Lehnert et al., 2019).
3. Selected Ion Flow Tube Mass Spectrometry (SIFT-MS) is gaining traction for its capacity to analyze gases directly and instantaneously, without the need for extensive prior preparation. This gainful characteristic enables researchers to monitor VOC fluxes in real time, which is inherently advantageous for studying plant responses to stresses such as drought or pathogen invasion (Lin et al., 2021; Danner et al., 2012).

### 6.2 Field-deployable proximal sensing technologies

Field-deployable proximal sensing technologies include portable devices that facilitate immediate measurement of plant VOCs under natural conditions. These technologies are essential for real-time monitoring in various agricultural contexts.

- Portable Gas Analyzers equipped with miniaturized GC-MS or MS systems enable on-site VOC measurements, providing actionable data for farmers and researchers (Rane et al., 2024). Integrating these technologies with cloud computing capabilities allows for data sharing and immediate decision-making (Rayes et al., 2015).
- Spectroscopy-Based Sensors work through techniques such as near-infrared (NIR) or other spectroscopic measurements to assess plant health and VOC emissions *in situ*, offering complementary data on physiological stress responses (Rayes et al., 2015).

### 6.3 Drone- and satellite-based atmospheric sensing approaches

The advent of UAVs and satellite technologies for atmospheric monitoring represents a paradigm shift in ecology and agriculture, enabling comprehensive ecosystem assessments at both macro and micro scales.

• Drones equipped with high-resolution sensors can capture fine-scale measurements of plant emissions. According to several studies, UAVs have proven effective for mapping and quantifying VOC emissions over large areas, thus providing vital data for ecological modeling and agricultural management (Merlaud et al., 2017; Ponce‐Campos et al., 2023). Satellite-based sensing approaches enhance the ability to monitor plant health by allowing for extensive spatial coverage. Here, satellite data can be combined with drone imagery to yield an integrated understanding of plant stress dynamics across diverse landscapes (Gadhwal and Sharda, 2023). This synergy enhances the precision of monitoring systems, thus improving agricultural practices and ecosystem management.

Despite their value for broad-scale monitoring, most remote-sensing platforms do not measure plant VOCs directly, but instead infer stress states through correlated physiological or spectral proxies such as canopy temperature, reflectance indices, fluorescence, or structural change. This introduces several uncertainties because such signals are not uniquely attributable to VOC emissions and may also reflect water status, nutrient limitation, disease severity, canopy architecture, mixed-species composition, or background atmospheric conditions. In addition, scale mismatch between leaf-level VOC emission processes and canopy- or landscape-level remote observations complicates interpretation, especially where signals are spatially heterogeneous or temporally transient. Remote sensing is therefore best viewed at present as an indirect and complementary approach for identifying candidate stress patterns, rather than as a fully resolved method for VOC detection without concurrent validation against direct chemical measurements (Oerke, 2020; Zhang et al., 2025).

### 6.4 Biosensors and wearable plant-monitoring systems

Biosensors and wearable technologies have emerged as novel approaches to monitor plant health through VOC detection.

- Wearable Sensors equipped with biosensitive materials can continuously monitor physiological indicators and VOC emissions, enabling real-time evaluation of plant health (Hasan et al., 2019). These devices differ from traditional methods by providing growers with immediate, direct feedback, thereby fostering better management practices in horticulture (Barrile et al., 2022).
- Colorimetric Sensors developed for specific VOCs can signal changes in plant health due to stress, allowing for non-invasive monitoring techniques that could reduce waste and labor in agricultural settings (Hossain et al., 2024).

### 6.5 Advances in continuous real-time monitoring systems

Recent developments in continuous monitoring systems incorporate advanced data analysis techniques, enabling automated evaluation of plant health through VOC emissions.

- Integration of remote sensing technologies along with data analytics has enabled a more nuanced understanding of plant health. The application of machine learning algorithms, paired with real-time data from instruments such as GC-MS and PTR-MS, enables predictive analytics of plant responses to stressors (Perez et al., 2015; Rane et al., 2024).
- Eddy covariance methods equipped with PTR-MS facilitate the measurement of VOC fluxes over broader ecological systems, enhancing the understanding of ecological dynamics and biogenic emissions on a larger scale (Acton et al., 2016).

Technological advancements now enable the detection of VOC emissions across laboratory, field, and atmospheric scales. **Table 4** summarizes major sensing platforms and their operational scales. The use of both laboratory-based and field-deployable technologies for monitoring plant volatile organic compounds is crucial for agricultural sustainability, plant physiology research, and ecosystem monitoring. Through a combination of traditional mass spectrometry and modern remote sensing techniques, researchers are now better equipped to understand the intricate relationships between plant responses to environmental stresses and the VOCs they emit.

**Table 4:** Technologies for detection and monitoring of plant volatile stress signals.

Technology category	Key instruments	Measurement scale	Strengths	Limitations
Laboratory analytical systems	GC-MS, PTR-MS, and SIFT-MS	Molecular/tissue level	High accuracy, and compound identification	Limited field applicability
Proximal sensing systems	Portable gas analyzers	Field/crop level	Real-time monitoring	Calibration variability
Remote sensing platforms	UAV-mounted sensors and satellites	Landscape/ecosystem level	Large-scale monitoring	Signal resolution challenges
Biosensors/wearable plant sensors	Colorimetric sensors and nanosensors	Individual plant level	Continuous monitoring	Limited compound specificity

## 7 Data integration, artificial intelligence, and predictive environmental monitoring

In the context of agriculture and ecosystem management, integrating data, artificial intelligence (AI), and machine learning (ML) technologies offers unprecedented potential to enhance our understanding of plant responses to stress. There are multifaceted approaches that may be employed to integrate these technologies for predictive environmental monitoring, focusing on the recognition of VOC patterns, multi-omics for stress detection, digital twins of physiological systems, decision-support systems, and scaling predictive stress detection from agricultural fields to larger ecosystems.

### 7.1 Machine learning approaches for VOC pattern recognition

Machine learning methodologies have emerged as a significant asset in recognizing, analyzing, and predicting plant VOC emissions in response to various stressors. Algorithms such as deep learning models are increasingly used to identify plant stress patterns from VOC and remote-sensing datasets, including convolutional neural networks (CNNs), which automatically learn hierarchical spatial features from high-dimensional inputs such as hyperspectral imagery, thermal images, and sensor arrays, and recurrent neural networks (RNNs), which capture temporal dependencies in sequential data and are therefore especially useful for tracking dynamic changes in plant stress signals over time (Jiang and Li, 2020; Kattenborn et al., 2021). CNNs and RNNs excel in processing complex datasets generated from environmental monitoring and remote sensing, enabling the identification of stress patterns that may not be readily observable with traditional methods (Buchha, 2025; Alphonse et al., 2025; Saygıner, 2025). The application of these technologies is exemplified by studies in which machine learning algorithms achieved remarkable accuracy in classifying plant health based on VOC emissions (Kayusi et al., 2025; Bianchi and Putro, 2024). As data continues to flood in from connected sensors and IoT devices, ML algorithms can uncover hidden patterns, providing timely alerts on plant stress and facilitating proactive management strategies.

### 7.2 Multi-omics integration for stress detection

Multi-omics approaches integrate data from genomics, transcriptomics, proteomics, and metabolomics to provide a holistic understanding of plant stress responses. By combining these diverse datasets, researchers can identify critical biomarkers that correlate with VOC emissions during stress events. This integrated approach enables more efficient detection and monitoring of stress conditions, thereby enhancing our predictive capabilities for plant health (Tripathi et al., 2025; VidhyaJanani et al., 2025). Machine learning plays a crucial role in this integration, as it helps to process and analyze the vast amounts of data generated by omics technologies (Reddy et al., 2024). In particular, neural networks are used to correlate various biological interactions and metabolomic changes with specific VOCs emitted by plants, leading to comprehensive stress-detection models. Predictive analytics derived from multi-omics data can inform decision-making in agricultural practices (Farooq and Manocha, 2023).

Current computational frameworks for integrating VOC data with multi-omics and AI typically combine multivariate statistics, supervised machine learning, pathway-based integration, and network-oriented systems biology approaches rather than relying on a single standardized pipeline. Commonly used methods include PCA, PLS-DA, correlation- and association-based integration, random forests, support vector machines, and deep-learning architectures such as CNNs and RNNs for high-dimensional pattern recognition, while pathway databases and network visualizations help link VOC features with transcriptomic, proteomic, and metabolomic responses (Jamil et al., 2020; Eicher et al., 2020). Emerging sensor-fusion and digital-twin approaches further extend these frameworks by integrating VOC measurements with physiological, environmental, and imaging data streams. However, their broader application remains limited by several challenges, including heterogeneity in data structure across instruments and omics layers, dimensionality imbalance, missing metadata, variable preprocessing pipelines, and limited model interpretability. These issues make it difficult to distinguish biologically meaningful signals from statistical associations and to transfer trained models reliably across species, stress contexts, and environments (Jamil et al., 2020; Pinu et al., 2019; Eicher et al., 2020).

### 7.3 Digital twins of plant physiological systems

Digital twins, which involve creating virtual representations of physical systems, have been increasingly applied to plant physiological systems to improve stress management strategies. These models integrate real-time data from environmental sensors, satellite imagery, and plant health diagnostics to simulate how plants respond to different stressors (Alavi and King, 2020; Semeraro et al., 2024; Kayusi et al., 2025). The predictive models achieved through these systems enable the identification of critical thresholds at which plants begin to exhibit stress behaviors, thus informing intervention strategies to mitigate stress impacts (Saygıner, 2025). Furthermore, integrating digital twins with advanced machine learning methods enables personalizing agricultural practices based on predictions derived from large datasets, optimizing resource use while reducing waste (Buchha, 2025; Rane et al., 2024).

### 7.4 Real-time decision-support systems for agriculture and conservation

Implementing real-time decision-support systems (DSS) represents a forward-thinking approach to managing agricultural landscapes and conserving natural ecosystems. These systems synthesize data from various sources, including remote sensing and IoT sensors, to derive actionable insights that inform farmers and conservationists about the current health of crops and surrounding environments (Alam et al., 2024; Rana and Bhambri, 2024). AI-driven models enhance the capabilities of decision-support systems by providing predictive analytics that identify potential risks and opportunities in agricultural landscapes by analyzing VOC patterns, environmental conditions, and other ecological variables. Machine learning models can accurately forecast pest outbreaks and stress events, enabling proactive management to increase productivity and sustainability in farming practices (Saygıner, 2025; Szramowiat-Sala, 2023).

### 7.5 Scaling predictive stress detection from fields to ecosystems

Scaling predictive stress detection translates insights from individual crops into broader ecosystems. This requires an understanding of how stress signals and agricultural practices affect not only the target plants but also the surrounding biodiversity and ecosystem functionality (Bianchi and Putro, 2024; Ojadi et al., 2023). The integration of machine learning with geospatial analysis enhances the ability to monitor and predict ecological changes at multiple scales (Saygıner, 2025; Reddy et al., 2024).

AI methodologies can analyze landscape-level data to reveal interactions among agricultural practices, pest species, and plant health, supporting conservation goals amid intensive agriculture (Kazanskiy et al., 2025). Employing deep learning and machine learning in this context can empower stakeholders to make informed decisions that impact both crop yield and ecosystem health. The combination of machine learning, multi-omics approaches, and digital innovation offers promising pathways to tackle the challenges posed by modern agriculture and climate change, ensuring sustainable practices that benefit both food production and biodiversity conservation.

## 8 Applications of plant volatile stress signals for climate resilience and ecosystem management

The utilization of plant VOCs has numerous implications across various fields such as agriculture, ecosystem management, and climate resilience. In applied agricultural and environmental systems, plant VOCs serve as operational volatile stressometers, providing actionable early-warning signals that support precision stress detection, adaptive crop management, and resilience-based ecosystem decision-making. Recent advances in understanding the signaling pathways underlying these compounds have enabled the development of innovative applications to detect stress responses in plants, optimize agricultural practices, and monitor ecosystem health.

### 8.1 Early-warning systems for crop stress detection

Early-warning systems that use VOC emissions can provide timely alerts on physiological stress in crops. Such systems are underpinned by studies demonstrating that different stressors, such as herbivory and environmental stress, activate distinct VOC profiles in plants, which can be monitored using gas chromatography-mass spectrometry (Mostafa et al., 2022a; Midzi et al., 2022). For example, it has been shown that specific VOCs, such as green leaf volatiles, are released following tissue damage and can serve as indicators of plant health (Fernández-Espinosa et al., 2022; Mostafa et al., 2022b). The implementation of sensors based on metal-organic frameworks has been proposed for the real-time detection of these compounds, enhancing early warning capabilities in agricultural settings (Jaishi et al., 2024).

**Figure 5:**
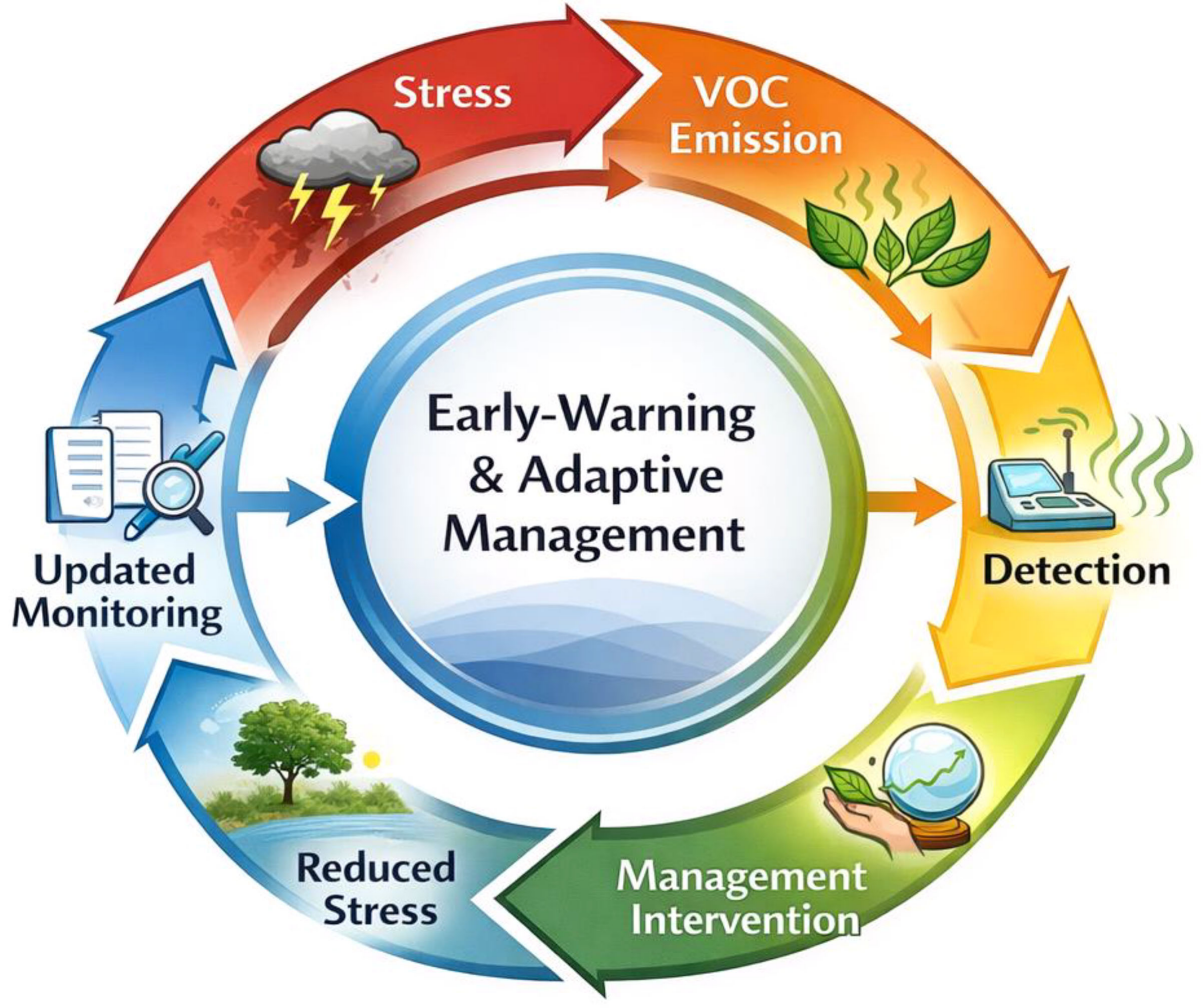
VOC-based early-warning and adaptive ecosystem management loop.

A notable advancement in this area is the development of integrated sensing systems that can correlate environmental and plant physiological data to predict stress outcomes. For instance, frameworks like the KAI2-mediated signaling pathways highlight the complex interactions between plant emissions and stress responses, further opening avenues for smart agriculture (Stirling et al., 2024). Integrating these technologies in crop management can lead to increased yields, reduced loss, and ultimately greater food security. **Figure 5** is a conceptual framework illustrating the circular feedback system through which stress-induced VOC emissions function as early-warning signals in ecosystems. Environmental or biotic stress triggers VOC release, which is detected through monitoring technologies and analytical platforms to enable predictive assessment of ecosystem risk. These predictions guide targeted management interventions aimed at reducing stress intensity and restoring system stability. Continuous post-intervention monitoring updates detection and prediction systems, creating an adaptive management loop that enhances resilience, improves decision-making, and supports proactive ecosystem management.

### 8.2 Precision agriculture and adaptive irrigation systems

Precision agriculture leverages IoT technology to enhance irrigation strategies tailored to crop demands (Jusman et al., 2024). The use of VOCs, particularly those associated with water stress, can improve the adaptability of irrigation systems, ensuring that plants receive optimal water supply under varying climatic conditions. For example, studies have indicated that understanding the volatile profiles emitted in response to drought stress can inform irrigation practices, allowing for more efficient water use (Tiwari et al., 2017).

Adaptive irrigation practices can substantially lower the risk of over- or under-watering crops, thereby optimizing nitrogen and other nutrient inputs while minimizing environmental impacts (Song et al., 2021). Research into the interaction between VOCs and phytohormones, such as abscisic acid (ABA), is crucial, as it aids in integrating physiological responses into adaptive irrigation strategies (Koramutla et al., 2021). Ongoing research is enhancing the sophistication of these systems to not only consider plant health but also regional climatic variations.

### 8.3 Forest health monitoring and wildfire risk detection

Monitoring forest health through VOC emissions provides critical insights into the ecological changes driving increased wildfire risk. Remote sensing technologies, which can detect changes in VOC profiles from trees, can serve as advanced tools for assessing forest health (Dudareva et al., 2013; Fernández-Espinosa et al., 2022). The emission of specific terpenes has been linked to stress responses in trees, signaling the potential onset of disease or pest invasions that could degrade forest integrity (Midzi et al., 2022). In this context, forest management practices could be refined by integrating VOC detection systems with predictive modeling techniques. By enabling real-time monitoring of forest conditions, these systems can help implement fire prevention measures and ecological rehabilitation practices, driven by data-driven decision-making (Dudareva et al., 2013; Fernández-Espinosa et al., 2022; Mostafa et al., 2022). The deployment of such technologies is integral to enhancing the resilience of forest ecosystems against climate change impacts.

To strengthen applied relevance, it is useful to highlight that VOC-based monitoring is already moving beyond conceptual promise into pilot and proof-of-concept implementation contexts. For example, green leaf volatile detection platforms and portable sensing systems have been explored for early crop stress identification, enabling detection of plant physiological disturbance before visible symptoms fully develop. Field-deployable monitoring approaches that combine VOC sensing with environmental or spectral data are also being tested as part of adaptive decision-support systems for crop management and ecosystem surveillance. In forestry and landscape contexts, VOC-informed stress assessment is increasingly discussed alongside remote sensing and predictive analytics as a complementary tool for detecting canopy stress, disturbance, and potential wildfire-linked deterioration. Although many of these efforts remain at the pilot or pre-operational stage, they illustrate the growing feasibility of translating VOC-based stress signaling into real-world monitoring and management workflows.

### 8.4 Biodiversity conservation and ecosystem disturbance tracking

VOCs play a pivotal role in plant-plant communication and can inform conservation efforts by indicating changes in resource availability that impact biodiversity (Midzi et al., 2022; Mostafa et al., 2022). By monitoring stress-induced volatile emissions, researchers can track shifts in plant community health and composition, thereby guiding restoration efforts and ecological interventions (Corrochano et al., 2016; Midzi et al., 2022). The application of molecular techniques to analyze the response of different species to VOC cues can reveal insights into their ecological relationships and functional roles within an ecosystem (Midzi et al., 2022). Future directions in this research area could include establishing comprehensive monitoring networks that integrate VOC measurements with biodiversity indices. By correlating changes in VOC profiles to shifts in species interactions and ecosystem services, these networks could promote proactive conservation strategies adaptable to ongoing environmental changes (Midzi et al., 2022; Delory et al., 2016).

### 8.5 Environmental compliance and pollution monitoring

The coupling of VOC monitoring with environmental compliance is an emerging application that assesses air and soil quality in agricultural and non-agricultural contexts. The emission of VOCs can indicate the presence of pollutants, especially heavy metals and pesticides, providing critical information for environmental management (Kosoe and Ogwu, 2025; Afzal et al., 2024). The detection of specific VOC profiles associated with contaminated soils helps determine compliance with environmental regulations on soil health and pollutant levels (Fernández-Espinosa et al., 2022; Shukla et al., 2017).

Technological advances in VOC detection technology, including portable sensors, are providing opportunities for field-based monitoring of both agricultural productivity and environmental health (Jaishi et al., 2024; Ogwu and Izah, 2024). Ongoing research into integrating these technologies with analytical models to predict pollutant impacts will further enhance environmental compliance across sectors, including agriculture, forestry, and urban environments (Afzal et al., 2024; Mostafa et al., 2022). The applications of plant volatile stress signals represent a rapidly evolving field with substantial implications for agricultural resilience, ecosystem management, and environmental monitoring. As technological advancements enhance our capacity to measure and interpret these emissions, stakeholders across sectors can develop more integrated, data-driven approaches to address the challenges posed by climate change and ecological disruption. The integration of VOC detection systems into environmental monitoring frameworks provides new tools for climate-resilient agriculture, ecosystem management, and environmental governance. **Table 5** summarizes major application domains.

**Table 5:** Applications of VOC-based monitoring for climate-resilient management.

Application area	VOC-based function	Management benefits
Precision agriculture	Early crop stress detection	Yield stability and reduced losses
Forest monitoring	Tree stress and pest detection	Wildfire risk management
Biodiversity conservation	Ecosystem disturbance detection	Restoration planning
Environmental compliance	Pollution monitoring indicators	Regulatory decision support
Climate modeling	VOC flux incorporation	Improved ecosystem forecasting

## 9 Limitations, knowledge gaps, and standardization challenges in decoding plant volatile stress signals

The investigation of plant VOCs is pivotal for our understanding of plant responses to various environmental stressors. However, several limitations and challenges hinder progress in this area, particularly concerning knowledge gaps and standardization issues. There is an overlapping need for improved methodologies and standardized practices to enhance research outcomes in the field.

### 9.1 Variability in volatile emissions across species and environments

The study of VOC emissions reveals significant variability not only among species but also within individual species under different environmental conditions. For example, research indicates that stress-induced emissions can vary considerably based on genetic makeup, geographical location, and environmental context (Midzi et al., 2022; Toome et al., 2010). Midzi et al. (2022); emphasize that species such as willow exhibit differing VOC patterns when subjected to biotic stress, demonstrating diminished emissions under specific infections compared to healthy counterparts. This illustrates that emissions are not universally applicable across species, which complicates efforts to draw broad conclusions from individual studies. Moreover, Toome et al. (2010) examined the impact of pathogen infection on willow and found that certain volatiles were suppressed, challenging the general assumption that VOC emissions are uniformly activated in response to stress. The intricacies of these interactions necessitate a more nuanced understanding of how environmental factors such as light, temperature, and humidity can modulate VOC emissions, further complicating comparisons across studies (Midzi et al., 2022; Toome et al., 2010).

### 9.2 Signal interference and atmospheric transformation processes

Once released into the atmosphere, plant VOCs do not remain chemically static; they are rapidly shaped by atmospheric oxidation processes that can alter both their persistence and biological significance. Many VOCs react with major atmospheric oxidants, including ozone (O_3_), hydroxyl radicals (OH), and nitrate radicals (NO_3_), which shorten signal lifetime, modify blend composition, and generate secondary oxidation products with distinct ecological and chemical properties (Holopainen and Blande, 2013; Blande et al., 2014; Fuentes et al., 2013; Ogwu et al., 2024). These reactions are especially important for highly reactive compounds such as monoterpenes, sesquiterpenes, and green leaf volatiles, whose atmospheric residence times may decline sharply under polluted or high-oxidant conditions, thereby reducing the distance over which biologically meaningful signals can be transmitted (Laothawornkitkul et al., 2009; Blande et al., 2014). Consistent with this, field studies often report effective volatile-mediated signaling over relatively short distances, commonly on the order of tens of centimeters to about one meter, while oxidant levels near 80 ppb ozone have been shown in some systems to disrupt biologically meaningful signal discrimination (Heil, 2014; Fuentes et al., 2013; Li et al., 2016). In addition to signal loss, atmospheric transformation can change the relative ratios of compounds within a volatile blend, potentially disrupting host location, neighbor perception, and higher trophic interactions by decoupling emitted signals from those ultimately perceived by receiving organisms (Li et al., 2016; Conchou et al., 2019). Oxidative processing may also produce secondary compounds and aerosols that introduce new ecological effects, further complicating the interpretation of VOC emissions outside controlled environments (Loreto et al., 2014; Blande et al., 2014). Atmospheric chemistry should therefore be treated as a central filtering process that mediates the performance of plant VOCs as volatile stressometers, particularly when extrapolating from laboratory studies to agricultural fields, forest canopies, and heterogeneous landscapes where oxidant loads, turbulence, and background chemical mixtures vary substantially.

### 9.3 Scaling challenges in ecosystem-level interpretation

While detailed studies on individual plants yield valuable insights into VOC emissions, scaling these findings to the ecosystem level poses substantial challenges (Teshome et al., 2020). Trees and other long-lived species experience a range of stresses over their life cycles, often in combination, and the cumulative effects on VOC emissions may not align with predictions from single-stress tests. Teshome et al. (2020) emphasize the need for integrative approaches that account for interactions among multiple stressors, as the impacts of climate change increasingly lead to concurrent stresses that traditional studies fail to capture. This scaling dilemma points to gaps in understanding how plant stress responses contribute to broader ecological dynamics, including changes in community structure and nutrient cycling, thereby necessitating the development of scalable models that incorporate multi-stressor approaches (Teshome et al., 2020).

### 9.4 Data comparability and methodological standardization

A considerable barrier within the field of VOC research is the lack of methodological consistency, which limits data comparability and synthesis across studies. Variations in experimental setups, the types of controlled variables, and the techniques used for VOC collection and analysis significantly influence outcomes (Midzi et al., 2023). Standardization in the sampling and analytical methods is paramount to ensure reliable data. For instance, Tholl et al. (2021) underline the importance of adopting consistent sampling techniques combined with gas chromatography-mass spectrometry for VOC analysis, which facilitates more rigorous comparisons between studies. As VOCs play critical roles in communication among plants and in interactions with herbivores, establishing standardized protocols will enhance the accuracy of method comparisons and enable shared findings across different ecological and agricultural studies, ultimately contributing to a more coherent understanding of plant stress responses (Copolovici et al., 2014; Tholl et al., 2021).

A major source of inconsistency in plant VOC studies arises from variation in sampling protocols, including enclosure design, headspace residence time, adsorption materials, humidity control, and the timing of sample collection relative to stress onset. Additional variability is introduced by differences in calibration standards, instrument sensitivity, background subtraction, peak deconvolution, compound annotation, and downstream data-processing pipelines. Interoperability across major detection platforms also remains limited. GC-MS offers strong compound separation and identification but is less suited for continuous real-time monitoring, whereas PTR-MS provides high temporal resolution for on-line measurements but often relies on compound libraries and calibration assumptions rather than chromatographic separation. Biosensor-based systems offer portability and continuous *in situ* deployment but currently have more limited compound specificity and are more susceptible to cross-sensitivity and calibration drift. These differences complicate direct comparisons across studies and underscore the need for standardized protocols, harmonized reporting practices, and cross-platform validation frameworks (Lehnert et al., 2019).

### 9.5 Ethical, technological, and infrastructure barriers

The complex ethical landscape surrounding biobanking and the use of biological samples adds another dimension to the challenges faced in VOC research. Ethical considerations are paramount, especially in low- and middle-income countries (LMICs), where the scientific infrastructure may not be as robust as in high-income nations (Chatfield et al., 2020; Zawati et al., 2014). Regulatory frameworks differ across jurisdictions, complicating international collaborations and data sharing, which are essential for large-scale research efforts (Zawati et al., 2014; Chatfield et al., 2020). Technological advancements also remain a critical barrier. While existing equipment for VOC analysis is effective, access to cutting-edge technologies can be limited in many LMICs, further widening the gap in research capabilities and outcomes (Vodosin et al., 2021; Chatfield et al., 2020). Developing more accessible, cost-effective technologies for VOC detection will enhance research prospects in these regions, ultimately leading to more inclusive studies of plant responses to environmental stressors (Jamalzadegan et al., 2025).

## 10 Future directions: toward global plant volatile monitoring networks

The excavation of VOCs provides critical insights into plant responses to stressors ranging from climate change impacts to insect infestations. Transitioning from isolated studies to a comprehensive understanding of these emissions across ecological scales is essential for robust environmental monitoring and management. The future of plant volatile monitoring networks hinges on several key areas outlined below.

### 10.1 Integrated biosensing infrastructures

The establishment of an integrated biosensing infrastructure will significantly enhance our capacity to acquire real-time data on VOC emissions across diverse ecosystems. Recent advancements in gas-chromatographic ion mobility spectrometry have enabled heightened sensitivity and rapid assessment of VOCs emitted from plants under both controlled and natural conditions (Vautz et al., 2018; Tani and Mochizuki, 2021). Current methodologies often fall short in capturing the nuanced patterns of emissions in the field due to logistical constraints. Utilizing wireless sensor networks (WSNs) can facilitate continuous monitoring and enhance our understanding of large-scale ecological interactions by closely analyzing VOCs released in response to environmental changes (Manes et al., 2012). Research indicates that VOC analysis not only enhances monitoring capabilities but also contributes to our understanding of ecosystem dynamics. For instance, studies on plant emissions under varying stress conditions, including environmental and biotic stresses, showcase the diverse profiles of emitted compounds (Dewhirst et al., 2020; Muraoka et al., 2019). These developments underline the necessity for integrating diverse sensing technologies that may include both empirical data and remote sensing capabilities to provide a more comprehensive view of atmospheric and terrestrial interactions.

### 10.2 Climate-linked volatile emission modeling

Improved models that couple climate variables with VOC emissions form a critical foundation for predicting how ecosystems respond to climate-related changes. Current research highlights the direct relationship between temperature and VOC emissions, notably how alterations in climate parameters can lead to increased emissions from flora (Rinnan et al., 2020; 2024). Enhanced models must incorporate factors such as soil moisture and vegetation composition to provide a multifaceted understanding of emissions scenarios under climate stress (Muraoka et al., 2019; Mäki, 2019; Rinnan, 2024). For instance, applying biogeochemical models that simulate soil-plant-atmosphere interactions can enhance predictions of the role of VOC emissions in broader climate dynamics. By refining parameters to include the specific VOC profiles of various plant species and their responses to abiotic stressors, improved flux models can significantly impact climate change research (Jardine et al., 2010; Tani and Mochizuki, 2021; Rinnan, 2024). This approach can help scientists anticipate shifts in atmospheric chemistry and their effects on climate.

### 10.3 Coupling plant stress signals with earth-system monitoring

Integrating plant stress signals into Earth-system monitoring presents a novel pathway to better understand connectivity across biogeochemical cycles. Given that VOCs are biogenic markers of stress that can indicate ecosystem health, combining these emissions data with existing Earth observation systems represents a significant advancement in environmental monitoring (Ghirardo et al., 2020; Mäki, 2019). Vital interactions can be examined closely, fostering a more in-depth understanding of plant-environment dynamics and their associated feedback mechanisms. Research demonstrates that VOCs can serve as indicators of ecosystem changes, reflecting shifts in vegetative health and stress levels (Dewhirst et al., 2020; Rinnan, 2024). By pairing this data with satellite-based surveillance and terrestrial measurements, comprehensive monitoring frameworks can be designed that better predict how terrestrial configurations influence atmospheric compositions, carbon cycles, and overall ecosystem resilience (Shugart et al., 2020).

### 10.4 Policy relevance and environmental governance implications

The ramifications of enhanced understanding and monitoring of plant VOC emissions extend into policy and environmental governance. Knowledge gained from these initiatives could directly inform regulations governing air quality and biodiversity conservation. Policies aimed at mitigating the adverse effects of climate-driven emissions would benefit from robust, data-backed frameworks that effectively assess the ecological impacts of plant stress signals (Dewhirst et al., 2020; Zamora et al., 2017; Franklin et al., 2016). Indeed, the establishment of monitoring networks informs various stakeholders about effective policies for land management, conservation, and emissions reduction. As the scientific community links VOC emissions to climate stressors, actionable insights can guide environmental policy and support sustainable practices in agriculture and ecosystem management (Paudel et al., 2021; Sharaf et al., 2023; Franklin et al., 2016).

### 10.5 Vision for next-generation environmental intelligence systems

The future prospects of environmental intelligence systems hinge upon leveraging advances in technology and data analytics. Artificial intelligence and machine learning could optimize the interpretation of complex, vast datasets acquired from biosensing infrastructures (Sharaf et al., 2023; Ma et al., 2022). This technological convergence enables more refined assessments of plant responses to climate change, with predictive capabilities that anticipate ecological shifts. Future systems require the integration of both ground-level measurements and remote sensing technologies to monitor spatial and temporal variations in VOCs across diverse ecosystems (Bringel and Couée, 2015; Zhang et al., 2025). Such integrated systems would not only strengthen our scientific understanding but would also facilitate proactive measures in environmental monitoring, ensuring that data-driven insights effectively operate within environmental governance frameworks (Mäki, 2019; Crombie et al., 2015; Francis et al., 2024). Plant volatile organic compounds are a cornerstone for understanding ecological responses to climate change and environmental stress. Advancing integrated monitoring networks, enhancing climate-linked modeling frameworks, and leveraging cutting-edge technology can have a profound impact on our understanding and management of ecosystems. Such comprehensive approaches will not only further ecological research but will also play a vital role in shaping policies aimed at environmental sustainability and resilience.

## 11 Conclusion

Plant volatile stress signals represent a unifying biochemical interface connecting molecular plant physiology, ecological communication networks, and atmospheric processes. Evidence across disciplines demonstrates that volatile emissions function not merely as metabolic by-products but as highly structured information systems that regulate plant defense, coordinate community-level responses, and influence ecosystem–atmosphere feedbacks. From intracellular transcriptional regulation to landscape-scale volatile fluxes, these chemical signals provide early indicators of environmental disturbance and adaptive plant responses, positioning VOCs as critical biomarkers of ecosystem health (Midzi et al., 2023).

Technological progress in analytical chemistry, biosensing, remote sensing, and artificial intelligence has significantly enhanced the capacity to detect and interpret volatile emissions, enabling real-time monitoring of plant stress and the development of predictive decision-support systems for agriculture, forestry, and environmental governance (Ederli et al., 2021). However, realizing the full potential of VOC-based monitoring requires overcoming key limitations related to methodological standardization, cross-species variability, atmospheric transformation processes, and the integration of multi-scale datasets. Coordinated global monitoring initiatives, interoperable sensor infrastructures, and climate-linked emission modeling frameworks will be essential for translating plant volatile signals into operational environmental intelligence systems. The integration of plant volatile monitoring with Earth-system observation platforms offers a powerful opportunity to advance climate resilience strategies, early-warning agricultural systems, and biodiversity conservation programs. By bridging molecular plant biology, ecological modeling, and environmental data science, next-generation research on plant volatile stress signaling can provide the scientific foundation for adaptive ecosystem management capable of responding proactively to accelerating environmental change.

## References

[B1] Acton W. Schallhart S. Langford B. Valach A. Rantala P. Fares S. . (2016). Canopy-scale flux measurements and bottom-up emission estimates of volatile organic compounds from a mixed oak and hornbeam forest in northern Italy. Atmos. Chem. Phys. 16, 7149–7170. doi: 10.5194/acp-16-7149-2016

[B2] Afzal M. Muhammad S. Tan D. Kaleem S. Khattak A. Wang X. . (2024). The effects of heavy metal pollution on soil nitrogen transformation and rice volatile organic compounds under different water management practices. Plants 13, 871. doi: 10.3390/plants13060871. PMID: 38592896 PMC10976017

[B3] Aggarwal A. Kadian N. Karishma K. Neetu N. Tanwar A. Gupta K. (2012). Arbuscular mycorrhizal symbiosis and alleviation of salinity stress. J. Appl. Natural Sci. 4, 144–155. doi: 10.31018/jans.v4i1.239

[B4] Aguirre N. M. Grunseich J. M. Lima A. F. Davis S. D. Helms A. M. (2023). Plant communication across different environmental contexts suggests a role for stomata in volatile perception. Plant Cell Environ. 46, 2017–2030. doi: 10.1111/pce.14601. PMID: 37165940

[B5] Alam M. Sohel A. Hasan K. Islam M. (2024). Machine learning and artificial intelligence in diabetes prediction and management: A comprehensive review of models. ITEJ 1, 107–124. doi: 10.70937/jnes.v1i01.41

[B6] Alavi N. King D. (2020). Evaluating the relationships of inter-annual farmland vegetation dynamics with biodiversity using multi-spatial and multi-temporal remote sensing data. Remote Sens. 12, 1479. doi: 10.3390/rs12091479. PMID: 30654563

[B7] Alphonse S. Rohit N. Yadeesh T. A. G. K. Karthinathan P. S. (2025). Deep learning models for intelligent IoT ecosystems. In Zhao J. (Ed.), AI in Advanced Systems, Robotics, and Healthcare ( IGI Global Scientific Publishing), pp. 37–72. doi: 10.4018/979-8-3373-7062-0.ch002

[B8] Aslam M. Waseem M. Jakada B. Okal E. Lei Z. Saqib H. . (2022). Mechanisms of abscisic acid-mediated drought stress responses in plants. Int. J. Mol. Sci. 23, 1084. doi: 10.3390/ijms23031084. PMID: 35163008 PMC8835272

[B9] Bacheva V. Madison I. Baldwin M. Baker J. Beilstein M. Call D. . (2025). Transdisciplinary collaborations for advancing sustainable and resilient agricultural systems. Global Change Biol. 31. doi: 10.1111/gcb.70142. PMID: 40197670 PMC11976515

[B10] Barrile V. Simonetti S. Citroni R. Fotia A. Bilotta G. (2022). Experimenting agriculture 4.0 with sensors: A data fusion approach between remote sensing, UAVs and self-driving tractors. Sensors 22, 7910. doi: 10.3390/s22207910. PMID: 36298261 PMC9611850

[B11] Bianchi O. Putro H. (2024). Artificial intelligence in environmental monitoring: Predicting and managing climate change impacts. Int. Trans. Artif. Intell. (Italic) 3, 85–96. doi: 10.33050/italic.v3i1.650

[B12] Blande J. Holopainen J. Niinemets Ü. (2014). Plant volatiles in polluted atmospheres: Stress responses and signal degradation. Plant Cell Environ. 37, 1892–1904. doi: 10.1111/pce.12352. PMID: 24738697 PMC4289706

[B13] Bohm K. Martín-Sánchez L. Garbeva P. (2017). Microbial volatiles: Small molecules with an important role in intra- and inter-kingdom interactions. Front. Microbiol. 8. doi: 10.3389/fmicb.2017.02484. PMID: 29312193 PMC5733050

[B14] Bringel F. Couée I. (2015). Pivotal roles of phyllosphere microorganisms at the interface between plant functioning and atmospheric trace gas dynamics. Front. Microbiol. 6. doi: 10.3389/fmicb.2015.00486. PMID: 26052316 PMC4440916

[B15] Buchha D. (2025). AI & ML for livestock welfare: Analysing behaviour and stress patterns for better care. Int. J. Sci. Res. Eng. Manage. 9, 1–9. doi: 10.55041/ijsrem46227

[B16] Chatfield K. Schroeder D. Guantai A. Bhatt K. Bukusi E. Odhiambo J. . (2020). Preventing ethics dumping: The challenges for Kenyan research ethics committees. Res. Ethics 17, 23–44. doi: 10.1177/1747016120925064

[B17] Chaudhry U. Gökçe Z. Gökçe A. (2022). Salt stress and plant molecular responses. doi: 10.5772/intechopen.101513

[B18] Chieb M. Gachomo E. (2023). The role of plant growth promoting rhizobacteria in plant drought stress responses. BMC Plant Biol. 23. doi: 10.1186/s12870-023-04403-8. PMID: 37626328 PMC10464363

[B19] Cofer T. Engelberth M. Engelberth J. (2018). Green leaf volatiles protect maize (Zea mays) seedlings against damage from cold stress. Plant Cell Environ. 41, 1673–1682. doi: 10.1111/pce.13204. PMID: 29601632

[B20] Conchou L. Lucas P. Meslin C. Proffit M. Staudt M. Renou M. (2019). Insect odorscapes: From plant volatiles to natural olfactory scenes. Front. Physiol. 10, 972. doi: 10.3389/fphys.2019.00972. PMID: 31427985 PMC6688386

[B21] Copolovici L. Kännaste A. Remmel T. Niinemets Ü. (2014). Volatile organic compound emissions from Alnus glutinosa under interacting drought and herbivory stresses. Environ. Exp. Bot. 100, 55–63. doi: 10.1016/j.envexpbot.2013.12.011. PMID: 29367790 PMC5777611

[B22] Corrochano L. Kuo A. Marcet‐Houben M. Polaino S. Salamov A. Villalobos‐Escobedo J. . (2016). Expansion of signal transduction pathways in fungi by extensive genome duplication. Curr. Biol. 26, 1577–1584. doi: 10.1016/j.cub.2016.04.038. PMID: 27238284 PMC5089372

[B23] Cortleven A. Leuendorf J. Frank M. Pezzetta D. Bolt S. Schmülling T. (2019). Cytokinin action in response to abiotic and biotic stresses in plants. Plant Cell Environ. 42, 998–1018. doi: 10.1111/pce.13494. PMID: 30488464

[B24] Crombie A. Khawand M. Rhodius V. Fengler K. Miller M. Whited G. . (2015). Regulation of plasmid‐encoded isoprene metabolism in Rhodococcus. Environ. Microbiol. 17, 3314–3329. doi: 10.1111/1462-2920.12793. PMID: 25727256 PMC4676930

[B25] Cuaran J. León J. (2021). Crop monitoring using unmanned aerial vehicles: A review. Agric. Rev. 42 (2), 121–132. doi: 10.18805/ag.r-180

[B26] Danner H. Samudrala D. Cristescu S. Dam N. (2012). Tracing hidden herbivores: Time-resolved non-invasive analysis of belowground volatiles by proton-transfer-reaction mass spectrometry (PTR-MS). J. Chem. Ecol. 38, 785–794. doi: 10.1007/s10886-012-0129-3. PMID: 22592334 PMC3375075

[B27] Das D. Kashtoh H. Panda J. Rustagi S. Mohanta Y. K. Singh N. . (2025). From hormones to harvests: a pathway to strengthening plant resilience for achieving sustainable. 10.3390/plants14152322 PMC12348992 40805671

[B28] Davison B. Brunner A. Ammann C. Spirig C. Jocher M. Neftel A. (2008). Cut‐induced VOC emissions from agricultural grasslands. Plant Biol. 10, 76–85. doi: 10.1055/s-2007-965043. PMID: 17538867

[B29] Delory B. Delaplace P. Fauconnier M. Jardin P. (2016). Root-emitted volatile organic compounds: Can they mediate belowground plant-plant interactions? Plant Soil 402, 1–26. doi: 10.1007/s11104-016-2823-3. PMID: 30311153

[B30] Dewhirst R. Mortimer J. Jardine K. (2020). Do cell wall esters facilitate forest response to climate? Trends Plant Sci. 25, 729–732. doi: 10.1016/j.tplants.2020.05.011. PMID: 32600937

[B31] De Cesare F. Molinari F. Valentini R. Agresti A. Macagnano A. (2025). Development of a selective molecularly imprinted polymer composite electrospun nanofiber sensor for a multifunctional platform for monitoring fruit tree health. EGU General Assembly 2025, Vienna, Austria, 27 Apr–2 May 2025, EGU25–20724. doi: 10.5194/egusphere-egu25-20724

[B32] Du B. Haensch R. Alfarraj S. Rennenberg H. (2024). Strategies of plants to overcome abiotic and biotic stresses. Biol. Rev. 99, 1524–1536. doi: 10.1111/brv.13079. PMID: 38561998

[B33] Dudareva N. Klempien A. Mühlemann J. K. Kaplan I. (2013). Biosynthesis, function and metabolic engineering of plant volatile organic compounds. New Phytol. 198, 16–32. doi: 10.1111/nph.12145. PMID: 23383981

[B34] Durand M. Lintunen A. Ezhova E. (2025). Forest canopy interactions with aerosols: Important considerations in approaching future impacts and climate management. New Phytol. 249, 114–122. doi: 10.1111/nph.70636. PMID: 41069110 PMC12676069

[B35] Ederli L. Salerno G. Quaglia M. (2021). In the tripartite combination Botrytis cinerea–Arabidopsis–Eurydema oleracea, the fungal pathogen alters the plant–insect interaction via jasmonic acid signalling activation and inducible plant-emitted volatiles. J. Plant Res. 134, 523–533. doi: 10.1007/s10265-021-01273-9. PMID: 33738682 PMC8106584

[B36] Eicher T. Kinnebrew G. Patt A. Spencer K. Ying K. Ma Q. . (2020). Metabolomics and multi-omics integration: A survey of computational methods and resources. Metabolites 10, 202. doi: 10.3390/metabo10050202. PMID: 32429287 PMC7281435

[B37] Engelberth J. (2021). Green leaf volatiles: Airborne signals that protect against biotic and abiotic stresses. Biology and Life Sciences Forum, 4 (1), 101. doi: 10.3390/IECPS2020-08634

[B38] Escobar‐Bravo R. Schimmel B. Zhang Y. Wang L. Robert C. Glauser G. . (2024). Far‐red light increases maize volatile emissions in response to volatile cues from neighbouring plants. Plant Cell Environ. 47, 3979–3998. doi: 10.1111/pce.14995. PMID: 38872585

[B39] Fernández-Espinosa A. J. Peña-Heras A. Rossini-Oliva S. (2022). Atmospheric emissions of volatile organic compounds from a mine soil treated with sewage sludge and tomato plants (Lycopersicum esculentum L.). Int. J. Environ. Res. 16, 47. doi: 10.1007/s41742-022-00425-6. PMID: 30311153

[B40] Farag M. Zhang H. Ryu C. (2013). Dynamic chemical communication between plants and bacteria through airborne signals: Induced resistance by bacterial volatiles. J. Chem. Ecol. 39, 1007–1018. doi: 10.1007/s10886-013-0317-9. PMID: 23881442 PMC3738840

[B41] Farooq B. Manocha A. (2023). A comparative study of deep learning and traditional methods for environmental remote sensing. ITM Web Conferences 56, 3002. doi: 10.1051/itmconf/20235603002

[B42] Fits L. Memelink J. (2000). ORCA3, a jasmonate-responsive transcriptional regulator of plant primary and secondary metabolism. Science 289, 295–297. doi: 10.1126/science.289.5477.295. PMID: 10894776

[B43] Francis C. Rektor A. Valayil-Varghese T. McKibben N. Estrada I. Forbey J. . (2024). Laser-induced graphene gas sensors for environmental monitoring. Front. Chem. 12. doi: 10.3389/fchem.2024.1448205. PMID: 39544719 PMC11560773

[B44] Franklin J. Serra‐Diaz J. Syphard A. Regan H. (2016). Global change and terrestrial plant community dynamics. PNAS 113, 3725–3734. doi: 10.1073/pnas.1519911113. PMID: 26929338 PMC4833242

[B45] Frolova N. (2025). Gas chromatography–mass spectrometry (GC-MS) in the plant metabolomics toolbox: Sample preparation and instrumental analysis. Biomolecules 16, 16. doi: 10.3390/biom16010016. PMID: 41594558 PMC12838911

[B46] Frost C. Appel H. Carlson J. Moraes C. Mescher M. Schultz J. (2007). Within‐plant signalling via volatiles overcomes vascular constraints on systemic signalling and primes responses against herbivores. Ecol. Lett. 10, 490–498. doi: 10.1111/j.1461-0248.2007.01043.x. PMID: 17498148

[B47] Frost C. Mescher M. Dervinis C. Davis J. Carlson J. Moraes C. (2008). Priming defense genes and metabolites in hybrid poplar by the green leaf volatile cis‐3‐hexenyl acetate. New Phytol. 180, 722–734. doi: 10.1111/j.1469-8137.2008.02599.x. PMID: 18721163

[B48] Fuentes J. D. Roulston T. H. Zenker J. (2013). Ozone impedes the ability of a herbivore to find its host. Environ. Res. Lett. 8, 14048. doi: 10.1088/1748-9326/8/1/014048

[B49] Gadhwal M. Sharda A. (2023). Feasibility and accuracy assessment of UAV, aircraft, and satellite-based remote sensing for irrigation management using canopy temperature and NDVI. 12539. doi: 10.1117/12.2664334

[B50] Ghirardo A. Lindstein F. Koch K. Buegger F. Schloter M. Albert A. . (2020). Origin of volatile organic compound emissions from subarctic tundra under global warming. Global Change Biol. 26, 1908–1925. doi: 10.1111/gcb.14935. PMID: 31957145 PMC7078956

[B51] Gigot C. Ongena M. Fauconnier M. Muhovski Y. Wathelet J. Jardin P. . (2012). Optimization and scaling up of a biotechnological synthesis of natural green leaf volatiles using Beta vulgaris hydroperoxide lyase. Process Biochem. 47, 2547–2551. doi: 10.1016/j.procbio.2012.07.018. PMID: 38826717

[B52] Gorman Z. Tolley J. Koiwa H. Kolomiets M. (2021). The synthesis of pentyl leaf volatiles and their role in resistance to anthracnose leaf blight. Front. Plant Sci. 12. doi: 10.3389/fpls.2021.719587. PMID: 34512698 PMC8427672

[B53] Hamid N. Naeem-ul-Hassan M. Zainal Z. Ismail I. (2023). Persicaria minor F-box gene Pmf-box1 indirectly affects Arabidopsis thaliana LOX-HPL pathway for green leaf volatile production. Sains Malaysiana 52, 1649–1670. doi: 10.17576/jsm-2023-5206-04

[B54] Hasan U. Sawut M. Chen S. (2019). Estimating the leaf area index of winter wheat based on unmanned aerial vehicle RGB-image parameters. Sustainability 11, 6829. doi: 10.3390/su11236829. PMID: 30654563

[B55] Hashem C. Hochrinner J. Bürgler M. Rinnofner C. Pichler H. Winkler M. (2022). From linoleic acid to hexanal and hexanol by whole cell catalysis with a lipoxygenase, hydroperoxide lyase and reductase cascade in Komagataella phaffii. Front. Mol. Biosci. 9. doi: 10.3389/fmolb.2022.965315. PMID: 36579187 PMC9791951

[B56] Heil M. (2014). Herbivore-induced plant volatiles: targets, perception and unanswered questions. New Phytol. 204, 297–306. doi: 10.1111/nph.12977. PMID: 40046247

[B57] Holopainen J. K. Blande J. D. (2013). Where do herbivore-induced plant volatiles go? Front. Plant Sci. 4, 185. doi: 10.3389/fpls.2013.00185. PMID: 23781224 PMC3678092

[B58] Hossain O. Wang Y. Li M. Jamalzadegan S. Mohammad N. Alireza A. . (2024). A dual-functional needle-based VOC sensing platform for rapid vegetable quality examination. doi: 10.1101/2024.12.12.628229 40064055

[B59] Howard M. Bass E. Chautá A. Mutyambai D. Keßler A. (2021). Integrating plant-to-plant communication and rhizosphere microbial dynamics: ecological and evolutionary implications and a call for experimental rigor. ISME J. 16, 5–9. doi: 10.1038/s41396-021-01063-0. PMID: 34333553 PMC8692333

[B60] Hu Z. Shen Y. Shen F. Luo Y. Su X. (2009). Evidence for the signaling role of methyl jasmonate, methyl salicylate, and benzothiazole between poplar (Populus simonii × P. pyramidalis ‘Opera 8277’) cuttings. Trees 23, 1003–1011. doi: 10.1007/s00468-009-0342-z. PMID: 30311153

[B61] Hu F. Zhang Y. Guo J. (2023). Identification and characterization of lipoxygenase (LOX) genes involved in abiotic stresses in yellow horn. PLoS One 18 (10), e0292898. doi: 10.1371/el 37831731 PMC10575502

[B62] Hu X. Zhang Z. Xu P. Fu Z. Hu S. Song W. (2010). Multifunctional genes: the cross-talk among the regulation networks of abiotic stress responses. Biol. Plant 54, 213–223. doi: 10.1007/s10535-010-0039-6. PMID: 30311153

[B63] Isah T. (2019). Stress and defense responses in plant secondary metabolites production. Biol. Res. 52. doi: 10.1186/s40659-019-0246-3. PMID: 31358053 PMC6661828

[B64] Jain A. Sarsaiya S. Singh R. Gong Q. Wu Q. Shi J. (2024). Omics approaches in understanding the benefits of plant-microbe interactions. Front. Microbiol. 15. doi: 10.3389/fmicb.2024.1391059. PMID: 38860224 PMC11163067

[B65] Jaishi L. Ding W. Kittelson R. Tsow F. Xian X. (2024). Metal–organic frameworks (MOFs)-based piezoelectric-colorimetric hybrid sensor for monitoring green leaf volatiles. ACS Sens. 9, 6553–6562. doi: 10.1021/acssensors.4c02016. PMID: 39587870

[B66] Jamalzadegan S. Xu J. Shen Y. Mativenga B. Li M. Zare M. . (2025). Advancing wearable VOC sensors: a roadmap for sustainable agriculture. Chem & Bio Engineering. 2, 460–474. doi: 10.1021/cbe.5c00027 PMC12400122 40901578

[B67] Jamil I. N. Remali J. Azizan K. A. Nor Muhammad N. A. Arita M. Goh H.-H. . (2020). Systematic multi-omics integration (MOI) approach in plant systems biology. Front. Plant Sci. 11, 944. doi: 10.3389/fpls.2020.00944. PMID: 32754171 PMC7371031

[B68] Jardine K. Abrell L. Kurc S. Huxman T. Ortega J. Guenther A. (2010). Volatile organic compound emissions from Larrea tridentata. Atmos. Chem. Phys. 10, 12191–12206. doi: 10.5194/acp-10-12191-2010

[B69] Jiang Y. Li C. (2020). Convolutional neural networks for image-based high-throughput plant phenotyping: a review. Plant Phenomics 2020, 4152816. doi: 10.34133/2020/4152816. PMID: 33313554 PMC7706326

[B70] Jogawat A. Yadav B. Lakra N. Singh A. Narayan O. (2021). Crosstalk between phytohormones and secondary metabolites in the drought stress tolerance of crop plants: a review. Physiol. Plant 172, 1106–1132. doi: 10.1111/ppl.13328. PMID: 33421146

[B71] Jusman Y. Chamim A. Zaki A. Loniza E. Winiarti S. Ferdiansyah R. . (2024). Implementation of an IoT-based automated watering system for melon cultivation. Bio Web Conferences 137, 1014. doi: 10.1051/bioconf/202413701014. PMID: 24184215

[B72] Kang S. Yuan R. Pannone A. Li Y. Ravichandran H. Kim S. . (2026). Detection and monitoring of volatile organic compounds (VOCs) via the use of engineered chemical sensors: an underexplored tool for safeguarding crop health and quality. Phytofrontiers®. 6 (1), 5–22. doi: 10.1094/phytofr-05-25-0053-rvw

[B73] Kanjana N. Guo P. Deng Z. Ahmed M. A. Shah I. Zhang L. (2026). Microbial volatile organic compounds reshape plant hormonal networks and root herbivore defense. Curr. Plant.

[B74] Kattenborn T. Leitloff J. Schiefer F. Hinz S. (2021). Review on convolutional neural networks (CNN) in vegetation remote sensing. ISPRS J. Photogramm. Remote Sens. 173, 24–49. doi: 10.1016/j.isprsjprs.2020.12.010. PMID: 38826717

[B75] Kayusi F. Kasulla S. Malik S. Chavula P. (2025). Article context and technological integration: AI's role in climate change research. LatIA 3, 85. doi: 10.62486/latia202585

[B76] Kazanskiy N. Khabibullin R. Никоноров А. Khonina S. (2025). A comprehensive review of remote sensing and artificial intelligence integration. Sensors 25, 5965. doi: 10.3390/s25195965. PMID: 41094788 PMC12527102

[B77] Kazimírová V. Zezulová V. Krasňan V. Štefuca V. Rebroš M. (2021). Optimization of hydroperoxide lyase production for recombinant lipoxygenase pathway cascade application. Catalysts 11, 1201. doi: 10.3390/catal11101201. PMID: 30654563

[B78] Khalid M. Shakeel A. Razzaq H. Amjad I. Amjad N. (2025). Sustainable cotton production in the era of climate change: challenges and adaptive measures. JAB 3, 402–415. doi: 10.55627/agribiol.003.01.1265

[B79] Klein M. Stewart J. Porter S. Weedon J. Kiers E. (2022). Evolution of manipulative microbial behaviors in the rhizosphere. Evol. Appl. 15, 1521–1536. doi: 10.1111/eva.13333. PMID: 36330300 PMC9624083

[B80] Knieper M. Viehhauser A. Dietz K. (2023). Oxylipins and reactive carbonyls as regulators of the plant redox and reactive oxygen species network under stress. Antioxidants 12, 814. doi: 10.3390/antiox12040814. PMID: 37107189 PMC10135161

[B81] Koramutla M. Negi M. Ayele B. (2021). Roles of glutathione in mediating abscisic acid signaling and its regulation of seed dormancy and drought tolerance. Genes 12, 1620. doi: 10.3390/genes12101620. PMID: 34681014 PMC8535772

[B82] Kosoe E. A. Ogwu M. C. (2025). “ Sustainable urban planning and environmental management,” in Evaluating Environmental Processes and Technologies. Eds. Ogwu M. C. Chibueze Izah S. Environmental Science and Engineering Series ( Springer, Cham). doi: 10.1007/978-3-031-85327-2_14

[B83] Kumar N. Jayanthi P. Kempraj V. Ravindra M. Roy T. Verghese A. (2017). Herbivore induced plant volatiles represents a favorable host to onion thrips (Thrips tabaci). Indian J. Agric. Sci. 87 (3), 373–378. doi: 10.56093/ijas.v87i3.68751

[B84] Kutty N. Mishra M. (2023). Dynamic distress calls: volatile info chemicals induce and regulate defense responses during herbivory. Front. Plant Sci. 14. doi: 10.3389/fpls.2023.1135000. PMID: 37416879 PMC10322200

[B85] Łabańska M. Amsterdam S. Jenkins S. Clarkson J. Covington J. (2022). Preliminary studies on detection of Fusarium basal rot infection in onions and shallots using electronic nose. Sensors 22, 5453. doi: 10.3390/s22145453. PMID: 35891126 PMC9315870

[B86] Laothawornkitkul J. Taylor J. E. Paul N. D. Hewitt C. N. (2009). Biogenic volatile organic compounds in the Earth system. New Phytol. 183, 27–51. doi: 10.1111/j.1469-8137.2009.02859.x. PMID: 19422541

[B87] Lehnert A. Behrendt T. Ruecker A. Pohnert G. Trumbore S. (2019). Performance of SIFT-MS and PTR-MS in the measurement of volatile organic compounds at different humidities. 13 (7). doi: 10.5194/amt-2019-349

[B88] Li T. Blande J. D. Holopainen J. K. Aphalo P. J. (2016). Atmospheric transformation of plant volatiles disrupts host plant finding. Sci. Rep. 6, 33851. doi: 10.1038/srep33851. PMID: 27651113 PMC5030639

[B89] Lin G. Vadhwana B. Belluomo I. Boshier P. Španěl P. Hanna G. (2021). Cross platform analysis of volatile organic compounds using selected ion flow tube and proton-transfer-reaction mass spectrometry. J. Am. Soc Mass Spectrom. 32, 1215–1223. doi: 10.1021/jasms.1c00027. PMID: 33831301

[B90] Loreto F. Dicke M. Schnitzler J.-P. Turlings T. C. J. (2014). Plant volatiles and the environment. Plant Cell Environ. 37, 1905–1908. doi: 10.1111/pce.12369. PMID: 24811745

[B91] Luo R. Lun X. Gao R. Wang L. Yang Y. Su X. . (2025). A review of biogenic volatile organic compounds from plants: research progress and future prospects. Toxics 13, 364. doi: 10.3390/toxics13050364. PMID: 40423443 PMC12115729

[B92] Ma X. Zhu X. Xie Q. Jin J. Zhou Y. Luo Y. . (2022). Monitoring nature's calendar from space. Global Change Biol. 28, 7186–7204. doi: 10.1111/gcb.16436. PMID: 36114727 PMC9827868

[B93] Mäki M. (2019). Volatile organic compound fluxes from northern forest soils. Dissertationes Forestales 2019. doi: 10.14214/df.275

[B94] Manes G. Collodi G. Fusco R. Gelpi L. Manes A. (2012). A wireless sensor network for precise volatile organic compound monitoring. Int. J. Distrib. Sens. Netw. 8, 820716. doi: 10.1155/2012/820716

[B95] Mathivanan S. (2021). Abiotic stress-induced molecular and physiological changes and adaptive mechanisms in plants. doi: 10.5772/intechopen.93367

[B96] Maynard D. Chibani K. Schmidtpott S. Seidel T. Spross J. Viehhauser A. . (2021). Biochemical characterization of 13-lipoxygenases of *Arabidopsis thaliana*. Int. J. Mol. Sci. 22 (19), 10237. 34638573 10.3390/ijms221910237 PMC8508710

[B97] Merlaud A. Tack F. Constantin D. Georgescu L. Maes J. Fayt C. . (2018). The Small Whiskbroom Imager for atmospheric compositioN monitorinG (SWING) and its operations from an unmanned aerial vehicle (UAV) during the AROMAT campaign. Atmos. Meas. Tech. 11 (1), 551–567. doi: 10.5194/amt-11-551-2018

[B98] Midgley G. (2017). Plant physiological responses to climate and environmental change, 1-12. doi: 10.1002/9780470015902.a0003205.pub2

[B99] Midzi J. Jeffery D. Baumann U. Capone D. Rogiers S. Pagay V. (2023). Evidence of bi-directional volatile-mediated communication. Agronomy 13, 1747. doi: 10.3390/agronomy13071747. PMID: 30654563

[B100] Midzi J. Jeffery D. Baumann U. Rogiers S. Tyerman S. Pagay V. (2022). Stress-induced volatile emissions and signalling in inter-plant communication. Plants 11, 2566. doi: 10.3390/plants11192566. PMID: 36235439 PMC9573647

[B101] Mostafa S. Wang Y. Zeng W. Jin B. (2022a). Floral scents and fruit aromas: functions, compositions, biosynthesis, and regulation. Front. Plant Sci. 13. doi: 10.3389/fpls.2022.860157. PMID: 35360336 PMC8961363

[B102] Mostafa S. Wang Y. Zeng W. Jin B. (2022b). Plant responses to herbivory, wounding, and infection. Int. J. Mol. Sci. 23, 7031. doi: 10.3390/ijms23137031. PMID: 35806046 PMC9266417

[B103] Munné-Bosch S. Peñuelas J. Asensio D. Llusià J. (2004). Airborne ethylene may alter antioxidant protection and reduce tolerance of holm oak to heat and drought stress. Plant Physiol. 136, 2937–2947. doi: 10.1104/pp.104.050005. PMID: 15448201 PMC523356

[B104] Muraoka H. Maruya Y. Nagai S. (2019). Long-term and multidisciplinary research on carbon cycling. J. Geogr. 128, 129–146. doi: 10.5026/jgeography.128.129

[B105] Ngumbi E. Dady E. Calla B. (2022). Flooding and herbivory: the effect of concurrent stress factors on plant volatile emissions and gene expression in two heirloom tomato varieties. BMC Plant Biol. 22. doi: 10.1186/s12870-022-03911-3. PMID: 36396998 PMC9670554

[B106] Ninkovic V. Marković D. Rensing M. (2020). Plant volatiles as cues and signals in plant communication. Plant Cell Environ. 44, 1030–1043. doi: 10.1111/pce.13910. PMID: 33047347 PMC8048923

[B107] Oerke E.-C. (2020). Remote sensing of diseases. Annu. Rev. Phytopathol. 58, 225–252. doi: 10.1146/annurev-phyto-010820-012832. PMID: 32853102

[B108] Ogwu M. C. (2019). Towards sustainable development in Africa: the challenge of urbanization and climate change adaptation. In: Cobbinah P. B. Addaney M. (eds) The Geography of Climate Change Adaptation in Urban Africa.

[B109] Ogwu M. C. (2025). “ Science and theory of pollution: sources, pathways, effects and pollution credit,” in Evaluating Environmental Processes and Technologies. Eds. Ogwu M. C. Chibueze Izah S. Environmental Science and Engineering Series ( Springer, Cham). doi: 10.1007/978-3-031-85327-2_5

[B110] Ogwu M. C. Imarhiagbe O. Ikhajiagbe B. Osawaru M. E. (2024). “ Toward understanding the impacts of air pollution,” in Sustainable Strategies for Air Pollution Mitigation, vol. 133 . Eds. Ogwu M. C. Izah S. C. The Handbook of Environmental Chemistry ( Springer, Cham). doi: 10.1007/698_2024_1107

[B111] Ogwu M. C. Izah S. C. (2024). “ Air pollution and the sustainable development goals,” in Sustainable Strategies for Air Pollution Mitigation, vol. 133 . Eds. Ogwu M. C. Izah S. C. The Handbook of Environmental Chemistry (Cham: Springer). doi: 10.1007/698_2024_1118

[B112] Ogwu M. C. Izah S. C. (2026). “ Climate frontiers and social transformation,” in World-Systems Evolution and Global Futures (6330 Cham, Zug, Switzerland). doi: 10.1007/978-3-032-17639-4

[B113] Ojadi J. Onukwulu E. Odionu C. Owulade O. (2023). AI-driven predictive analytics for carbon emission reduction. IJMRGE 4, 948–960. doi: 10.54660/.ijmrge.2023.4.1.948-960

[B114] Palai G. Caruso G. Gucci R. D’Onofrio C. (2023). Water deficit before veraison is crucial in regulating berry VOCs concentration in Sangiovese grapevines. Front. Plant Sci. 14. doi: 10.3389/fpls.2023.1117572. PMID: 36890905 PMC9986437

[B115] Papazian S. Khaling E. Bonnet C. Lassueur S. Reymond P. Möritz T. . (2016). Central metabolic responses to ozone and herbivory affect photosynthesis and stomatal closure. Plant Physiol. 172, 2057–2078. doi: 10.1104/pp.16.01318. PMID: 27758847 PMC5100778

[B116] Paudel G. Adhikari S. Jojiju B. Adhikari R. Adhikar N. (2021). Impact of climate change on the ecosystem of the central Himalayas. Arch. Agric. Environ. Sci. 6, 360–366. doi: 10.26832/24566632.2021.0603015

[B117] Peñaflor M. Erb M. Robert C. Miranda L. Werneburg A. Dossi F. . (2011). Oviposition by a moth suppresses constitutive and herbivore-induced plant volatiles in maize. Planta 234, 207–215. doi: 10.1007/s00425-011-1409-9. PMID: 21509694

[B118] Perez M. Zech W. Donald W. (2015). Using unmanned aerial vehicles to conduct site inspections of erosion and sediment control practices and track project progression. Transportation Res. Record: J. Transportation Res. Board 2528, 38–48. doi: 10.3141/2528-05

[B119] Pham Y. Beauchamp J. (2022). Analytical approaches for disease detection. In Haick H. (Ed.), Volatile biomarkers for human health: From nature to artificial senses ( The Royal Society of Chemistry, Chapter 16; pp. 284–322. doi: 10.1039/9781839166990-00284

[B120] Picazo-Aragonés J. Terrab A. Balao F. (2020). Plant volatile organic compounds evolution: Transcriptional regulation, epigenetics and polyploidy. Int. J. Mol. Sci. 21, 8956. doi: 10.3390/ijms21238956. PMID: 33255749 PMC7728353

[B121] Pinto D. M. Blande J. D. Souza S. R. Nerg A.-M. Holopainen J. K. (2010). Plant volatile organic compounds (VOCs) in ozone (O_3_) polluted atmospheres: The ecological effects. J. Chem. Ecol. 36, 22–34. doi: 10.1007/s10886-009-9732-3. PMID: 20084432

[B122] Pinu F. R. Beale D. J. Paten A. M. Kouremenos K. Swarup S. Schirra H. J. . (2019). Systems biology and multi-omics integration: Viewpoints from the metabolomics research community. Metabolites 9, 76. doi: 10.3390/metabo9040076. PMID: 31003499 PMC6523452

[B123] Ponce‐Campos G. McClaran M. Heilman P. Gillan J. (2023). UAV and satellite-based sensing to map ecological states at the landscape scale. Open J. Ecol. 13, 560–596. doi: 10.4236/oje.2023.138035

[B124] Portal-González N. Wang W. He W. Santos R. (2025). Engineering plant holobionts for climate-resilient agriculture. ISME J. 19. doi: 10.1093/ismejo/wraf158. PMID: 40748243 PMC12381762

[B125] Prugh L. Deguines N. Grinath J. Suding K. Bean W. Stafford R. . (2018). Ecological winners and losers of extreme drought in California. Nat. Clim. Change 8, 819–824. doi: 10.1038/s41558-018-0255-1. PMID: 37880705

[B126] Qi Y. Pan J. Zhang Z. Xu J. (2024). Whole-cell one-pot biosynthesis of dodecanedioic acid from renewable linoleic acid. Bioresour. Bioprocess. 11. doi: 10.1186/s40643-024-00770-8. PMID: 38780695 PMC11116355

[B127] Qi J. Zhou G. Yang L. Erb M. Lü Y. Sun X. . (2011). The chloroplast-localized phospholipases D α4 and α5 regulate herbivore-induced direct and indirect defenses in rice. Plant Physiol. 157, 1987–1999. doi: 10.1104/pp.111.183749. PMID: 21984727 PMC3327179

[B128] Qian X. Xu X. Yu K. Zhu B. Lan Y. Duan C. . (2016). Varietal dependence of GLVs accumulation and LOX-HPL pathway gene expression in four Vitis vinifera wine grapes. Int. J. Mol. Sci. 17, 1924. doi: 10.3390/ijms17111924. PMID: 27886056 PMC5133920

[B129] Rana R. Bhambri P. (2024). Harnessing AI and machine learning for enhanced geospatial analysis. In Darwish D. Chemingui H. (Eds.), Recent Trends in Geospatial AI ( IGI Global Scientific Publishing), pp. 27–72. doi: 10.4018/979-8-3693-8054-3.ch002

[B130] Rane N. Kaya Ö. Rane J. (2024). Artificial intelligence, machine learning, and deep learning technologies as catalysts for industry 4.0, 5.0, and society 5.0. doi: 10.70593/978-81-981271-8-1_1

[B131] Rani A. Tokas J. (2020). Transcriptional regulation of proline biosynthesis. Int. J. Advanced Res. 8, 67–73. doi: 10.21474/ijar01/10436. PMID: 42073092

[B132] Rasmann S. Turlings T. (2007). Simultaneous feeding by aboveground and belowground herbivores attenuates plant-mediated attraction of their respective natural enemies. Ecol. Lett. 10, 926–936. doi: 10.1111/j.1461-0248.2007.01084.x. PMID: 17845293

[B133] Rayes Y. E. I. Beyrouthy M. E. I. Azzi D. E. I. (2015). Precision viticulture: The merging of an old concept with new technologies. Adv. Crop. Sci. Tech. 3, e132. doi: 10.4172/2329-8863.1000e132

[B134] Raza W. Wei Z. Jousset A. Shen Q. Friman V. (2021). Extended plant metarhizobiome: Understanding volatile organic compound signaling in plant-microbe metapopulation networks. mSystems 6. doi: 10.1128/msystems.00849-21. PMID: 34427518 PMC8407245

[B135] Reddy S. Manjunath D. Jahnavi S. Nandini C. (2024). A comprehensive review of machine learning approaches in livestock health monitoring. JBDTBA 3, 11–19. doi: 10.46610/jbdtba.2024.v03i03.002

[B136] Rinnan R. (2024). Volatile organic compound emissions in the changing arctic. Annu. Rev. Ecol. Evol. Syst. 55, 227–249. doi: 10.1146/annurev-ecolsys-102722-125156. PMID: 41139587

[B137] Rinnan R. Iversen L. Tang J. Vedel-Petersen I. Schollert M. Schurgers G. (2020). Separating direct and indirect effects of rising temperatures. PNAS 117, 32476–32483. doi: 10.1073/pnas.2008901117. PMID: 33257556 PMC7768730

[B138] Sardans J. Peñuelas J. (2021). Potassium control of plant functions: Ecological and agricultural implications. Plants 10, 419. doi: 10.3390/plants10020419. PMID: 33672415 PMC7927068

[B139] Sarsaiya S. Jain A. Singh R. Gong Q. Wu Q. Chen J. . (2025). Unveiling the rhizosphere microbiome of Dendrobium: mechanisms, microbial interactions, and implications for sustainable agriculture. Front. Microbiol. 16. doi: 10.3389/fmicb.2025.1531900. PMID: 39944638 PMC11814445

[B140] Saygıner C. (2025). AI-driven geospatial analysis in ecosystem management: Integration of machine learning methods. Turkish J. For. Sci. 9, 560–584. doi: 10.32328/turkjforsci.1704059

[B141] Semeraro T. Leggieri A. Calisi A. Scarano A. (2024). Increase of landscape ecosystem services generated by agrivoltaics systems. Matec Web Conferences 396, 16001. doi: 10.1051/matecconf/202439616001

[B142] Sharaf A. Nuc P. Ripl J. Alquicer G. Ibrahim E. Wang X. . (2023). Transcriptome dynamics in Triticum aestivum. Viruses 15, 689. doi: 10.3390/v15030689. PMID: 36992398 PMC10054045

[B143] Sharifi R. Ryu C. M. (2018). Revisiting bacterial volatile-mediated plant growth promotion: Lessons from the past and objectives for the future. Ann. Bot. 122, 349–358. doi: 10.1093/aob/mcy108. PMID: 29982345 PMC6110341

[B144] Sharifi R. Ryu C. (2020). Social networking in crop plants: Wired and wireless cross‐plant communications. Plant Cell Environ. 44, 1095–1110. doi: 10.1111/pce.13966. PMID: 33274469 PMC8049059

[B145] Shiojiri K. Kishimoto K. Ozawa R. Kugimiya S. Urashimo S. Arimura G. . (2006). Changing green leaf volatile biosynthesis in plants: An approach for improving plant resistance against both herbivores and pathogens. PNAS 103, 16672–16676. doi: 10.1073/pnas.0607780103. PMID: 17075049 PMC1636513

[B146] Shugart H. Foster A. Wang B. Druckenbrod D. Ma J. Lerdau M. . (2020). Gap models across micro- to mega-scales of time and space: examples of Tansley’s ecosystem concept. For. Ecosyst. 7. doi: 10.1186/s40663-020-00225-4. PMID: 38164791

[B147] Shukla A. Srivastava S. Suprasanna P. (2017). Genomics of metal stress-mediated signalling and plant adaptive responses in reference to phytohormones. Curr. Genomics 18, 512–522. doi: 10.2174/1389202918666170608093327. PMID: 29204080 PMC5684655

[B148] Song C. Yan J. Yang W. Yu Y. Zhang J. Liu Y. (2021). Research progress on the influence of irrigation methods on ammonia volatilization in farmland. IOP Conf. Series: Earth Environ. Sci. 647, 12170. doi: 10.1088/1755-1315/647/1/012170

[B149] Soriano K. McCormick A. (2020). Volatile emissions of six New Zealand fern species in response to physical damage and herbivory. N. Z. J. Ecol. 44. doi: 10.20417/nzjecol.44.5

[B150] Stirling S. Guercio A. Patrick R. Huang X. Bergman M. Dwivedi V. . (2024). Volatile communication in plants relies on a KAI2-mediated signaling pathway. Science 383, 1318–1325. doi: 10.1126/science.adl4685. PMID: 38513014

[B151] Stoy P. Trowbridge A. Siqueira M. Freire L. Phillips R. Jacobs L. . (2021). Vapor pressure deficit helps explain biogenic volatile organic compound fluxes from the forest floor and canopy of a temperate deciduous forest. Oecologia 197, 971–988. doi: 10.1007/s00442-021-04891-1. PMID: 33677772

[B152] Szramowiat-Sala K . (2023). Artificial intelligence in environmental monitoring: Application of artificial neural networks and machine learning for pollution prevention and toxicity measurements. Preprints.org. doi: 10.20944/preprints202307.1298.v1

[B153] Tamang B. Fukao T. (2015). Plant adaptation to multiple stresses during submergence and following desubmergence. Int. J. Mol. Sci. 16, 30164–30180. doi: 10.3390/ijms161226226. PMID: 26694376 PMC4691168

[B154] Tanaka M. Koeduka T. Matsui K. (2021). Green leaf volatile-burst in Selaginella moellendorffii. Front. Plant Sci. 12. doi: 10.3389/fpls.2021.731694. PMID: 34777416 PMC8578206

[B155] Tani A. Mochizuki T. (2021). Exchanges of volatile organic compounds between terrestrial ecosystems and the atmosphere. J. Agric. Meteorol. 77, 66–80. doi: 10.2480/agrmet.d-20-00025. PMID: 15923096

[B156] Teshome D. Zharare G. Naidoo S. (2020). The threat of the combined effect of biotic and abiotic stress. Front. Plant Sci. 11. doi: 10.3389/fpls.2020.601009. PMID: 33329666 PMC7733969

[B157] Tholl D. Hossain O. Weinhold A. Röse U. Wei Q. (2021). Trends and applications in plant volatile sampling and analysis. Plant J. 106, 314–325. doi: 10.1111/tpj.15176. PMID: 33506558

[B158] Tian J. Ma Z. Zhao K. Zhang J. Xiang L. Chen L. (2018). Transcriptomic and proteomic approaches to explore the differences in monoterpene and benzenoid biosynthesis between scented and unscented genotypes of wintersweet. Physiol. Plant 166, 478–493. doi: 10.1111/ppl.12828. PMID: 30216458

[B159] Tiwari S. Lata C. Chauhan P. Prasad V. Prasad M. (2017). A functional genomic perspective on drought signalling and its crosstalk with phytohormone-mediated signalling pathways in plants. Curr. Genomics 18, 469–482. doi: 10.2174/1389202918666170605083319. PMID: 29204077 PMC5684651

[B160] Tong X. Qi J. Zhu X. Mao B. Zeng L. Wang B. . (2012). The rice hydroperoxide lyase OsHPL3 functions in defense responses by modulating the oxylipin pathway. Plant J. 71, 763–775. doi: 10.1111/j.1365-313x.2012.05027.x. PMID: 22519706

[B161] Toome M. Randjärv P. Copolovici L. Niinemets Ü. Heinsoo K. Luik A. . (2010). Leaf rust induced volatile organic compounds signalling in willow. Planta 232, 235–243. doi: 10.1007/s00425-010-1169-y. PMID: 20419383

[B162] Tripathi A. Upadhyay P. Goel P. (2025). Neural networks for analyzing soil organic carbon storage. In Pandey H. Goel P. Balyan V. Yadav S. (eds) Advanced Systems for Monitoring Carbon Sequestration ( IGI Global Scientific Publishing), pp. 455–480. doi: 10.4018/979-8-3373-2091-5.ch019

[B163] Várallyay G. (2002). Assessment of environmental susceptibility/vulnerability of soils. Acta Agraria Debreceniensis 9, 62–74. doi: 10.34101/actaagrar/9/3563. PMID: 42065592

[B164] Vasseur-Coronado M. Vlassi A. Boulois H. Schuhmacher R. Parich A. Pertot I. . (2021). Ecological role of volatile organic compounds emitted by Pantoea agglomerans as interspecies and interkingdom signals. Microorganisms 9, 1186. doi: 10.3390/microorganisms9061186. PMID: 34072820 PMC8229667

[B165] Vautz W. Hariharan C. Weigend M. (2018). Smell the change. Ecol. Evol. 8, 4370–4377. doi: 10.1002/ece3.3990. PMID: 29760879 PMC5938450

[B166] VidhyaJanani J. Vigneshwaran G. Vikram N. Radhakrishnan D. Saranya R. Manojkumar R. . (2025). Artificial intelligence in water quality monitoring for sustainable resource management. Int. J. Environ. Sci. 11 (14s), 1528–1534. doi: 10.64252/wztgjj36

[B167] Vincenti S. Mariani M. Alberti J. Jacopini S. Caraffa V. Berti L. . (2019). Biocatalytic synthesis of natural green leaf volatiles using the lipoxygenase metabolic pathway. Catalysts 9, 873. doi: 10.3390/catal9100873. PMID: 30654563

[B168] Vodosin P. Jorgensen A. Mendy M. Kozlakidis Z. Caboux É. Zawati M. (2021). A review of regulatory frameworks governing biobanking. Biopreserv. Biobanking 19, 444–452. doi: 10.1089/bio.2020.0101. PMID: 33945303

[B169] Vwioko D. E. Okoekhian I. Ogwu M. C. (2018). Stress analysis of Amaranthus hybridus L. and Lycopersicon esculentum Mill. exposed to sulphur and nitrogen dioxide. Pertanika J. Trop. Agric. Sci. 41 (3), 1169–1191.

[B170] Wahab A. Muhammad M. Munir A. Abdi G. Zaman W. Ayaz A. . (2023). Role of arbuscular mycorrhizal fungi in regulating growth, enhancing productivity, and potentially influencing ecosystems under abiotic and biotic stresses. Plants 12, 3102. doi: 10.3390/plants12173102. PMID: 37687353 PMC10489935

[B171] Wang J. Song L. Gong X. Xu J. Li M. (2020). Functions of jasmonic acid in plant regulation and response to abiotic stress. Int. J. Mol. Sci. 21 (4), 1446. 32093336 10.3390/ijms21041446 PMC7073113

[B172] Wang L. Erb M. (2022). Volatile uptake, transport, perception, and signaling shape a plant’s nose. Essays Biochem. 66, 695–702. doi: 10.1042/EBC20210092. PMID: 36062590 PMC9528081

[B173] Wang B. Shuman J. Shugart H. Lerdau M. (2018). Biodiversity matters in feedbacks between climate change and air quality: a study using an individual‐based model. Ecol. Appl. 28, 1223–1231. doi: 10.1002/eap.1721. PMID: 29603469

[B174] Werner C. Wallrabe U. Christen A. Comella L. Dormann C. Göritz A. . (2024). ECOSENSE - Multi-scale quantification and modelling of spatio-temporal dynamics of ecosystem processes by smart autonomous sensor networks. Res. Ideas Outcomes 10. doi: 10.3897/rio.10.e129357

[B175] Xu Z. Pandey G. Ali Z. Zhang D. (2024). Editorial: Molecular basis of stress resistant signal transduction in plants: a biotechnological intervention to develop climate-resilient crops. Front. Plant Sci. 15. doi: 10.3389/fpls.2024.1356520. PMID: 38495371 PMC10940911

[B176] Zamora R. Pérez‐Luque A. Bonet F. (2017). Monitoring global change in high mountains. In Catalan J. Ninot J. Aniz M. (eds) High Mountain Conservation in a Changing World. Advances in Global Change Research, vol. 62. (Cham: Springer), 385–413. doi: 10.1007/978-3-319-55982-7_16

[B177] Zawati M. Knoppers B. Thorogood A. (2014). Population biobanking and international collaboration. Pathobiology 81, 276–285. doi: 10.1159/000357527. PMID: 25792216

[B178] Zhang C. Bäck J. Peñuelas J. Liu D. Filella I. Porcar-Castell A. . (2025). Connecting optical remote sensing of plant photosynthesis with biogenic volatile organic compound emissions. New Phytol. 248, 494–506. doi: 10.1111/nph.70504. PMID: 40887857 PMC12445812

[B179] Zhang L. Zhao M. Aikeremu F. Huang H. You M. Zhao Q. (2023). Involvement of three chemosensory proteins in perception of host plant volatiles in the tea green leafhopper, Empoasca onukii. Front. Physiol. 13, 1068543. doi: 10.3389/fphys.2022.1068543. PMID: 36685201 PMC9845707

[B180] Zheng X. Liu J. Wang X. (2025). Quorum signaling molecules: Interactions between plants and associated pathogens. Int. J. Mol. Sci. 26, 5235. doi: 10.3390/ijms26115235. PMID: 40508052 PMC12154563

